# Evidence and evidence gaps of laryngeal cancer surgery

**DOI:** 10.3205/cto000130

**Published:** 2016-12-15

**Authors:** Susanne Wiegand

**Affiliations:** 1 Department of Otolaryngology, Head & Neck Surgery, University Hospital of Leipzig, Germany

**Keywords:** laryngeal cancer, laryngectomy, open partial laryngectomy, transoral laser surgery, transoral robotic surgery

## Abstract

Surgical treatment of laryngeal cancer has been established for decades. In addition to total laryngectomy, which was first performed in 1873, a large number or organ preservation surgical techniques, like open partial laryngectomy, transoral laser microsurgery, and transoral robotic surgery have been developed. Studies on laryngeal cancer surgery are mainly retrospective case series and cohort studies. The evolution of chemoradiation protocols and their analysis in prospective randomized trials have led to an increasing acceptance of non-surgical treatment procedures. In addition to an improvement of prognosis, in recent years the preservation of function and maintenance of life quality after primary therapy of laryngeal cancer has increasingly become the focus of therapy planning. Significant late toxicity after chemoradiation has been identified as an important issue. This leads to a reassessment of surgical concepts and initiation of studies on laryngeal cancer surgery which was additionally stimulated by the advent of transoral robotic surgery in the US. Improving the evidence base of laryngeal cancer surgery by successful establishment of surgical trials should be the future goal.

## 1 Introduction

Especially in European countries, surgical therapy of laryngeal cancer has a long tradition. Despite the high rate of worldwide performed surgeries of laryngeal cancer and the partly long history of those interventions the scientific evaluation of the applied techniques is only sparse. 

Evidence-based medicine means the conscientious, explicit, and judicious use of current best evidence in making decisions about the care of individual patients. It encompasses the integration of individual clinical expertise and the best available external evidence from systematic research [1], [2]. The hierarchy of external evidence is based on the decreasing validity of study results. In Germany, evidence-based medicine is incorporated in social legislation. The best available evidence should be the base for healthcare management.

Evidence-based medicine is not limited to randomized controlled trials even if they are considered as gold standard of evidence-based medicine. In case no controlled studies were conducted for certain therapeutic measures or studies were not possible, the best available external evidence should be taken into account. It is estimated that only about 15–40% of all medical decisions are based on scientific evidence [3], [4]. On the other hand it is assumed that nearly half of all trials that are conducted worldwide are not published [5].

The lack of randomized controlled trials on surgical procedures is a problem that has been discussed since many years. The fact that randomized controlled trials on non-surgical treatment concepts have confirmed the effectiveness of primary radio(chemo)therapy for the treatment of laryngeal cancer, led to a modification of laryngeal cancer therapy. The possibilities of 3-dimensional radiation planning, changed fractionation schemes, and the combination with chemotherapy led to new aspects in the therapy of advanced laryngeal cancer. Regarding radiochemotherapy of laryngeal carcinomas, evidence level I is reported. Especially in the US, many departments therefore prefer primary radio(chemo)therapy to primary surgery.

A review on the effectiveness and efficiency of surgery is not only necessary in order to check its significance compared to non-operative therapy modalities. Also with regard to scarcer financial resources of the healthcare system a verification of the effectiveness and efficiency of therapeutic procedures will be needed in the future. The aim of this review is to assess the existing evidence of laryngeal cancer surgery and to identify possible evidence gaps.

## 2 Epidemiology of laryngeal cancer

Laryngeal cancer is the most frequently occurring malignant tumor of the head and neck. In 2010, about 3,230 men and 460 women developed malignomas of the larynx in Germany. This corresponds to an age-standardized incidence of around 6.0 new diseases per 100,000 male and 0.8 new diseases per 100,000 female inhabitants per year. Thus, in Germany 1 of 170 men and 1 of about 1,200 women develops laryngeal cancer during his/her life. In total, the incidence of laryngeal cancer in men decreases, especially regarding inhabitants younger than 50 years; the incidence for women is stable. Due to the higher life expectancy, the mean age of disease onset increases. At present, for females it is 63 years and for males 66 years [6].

**Figure 1:**
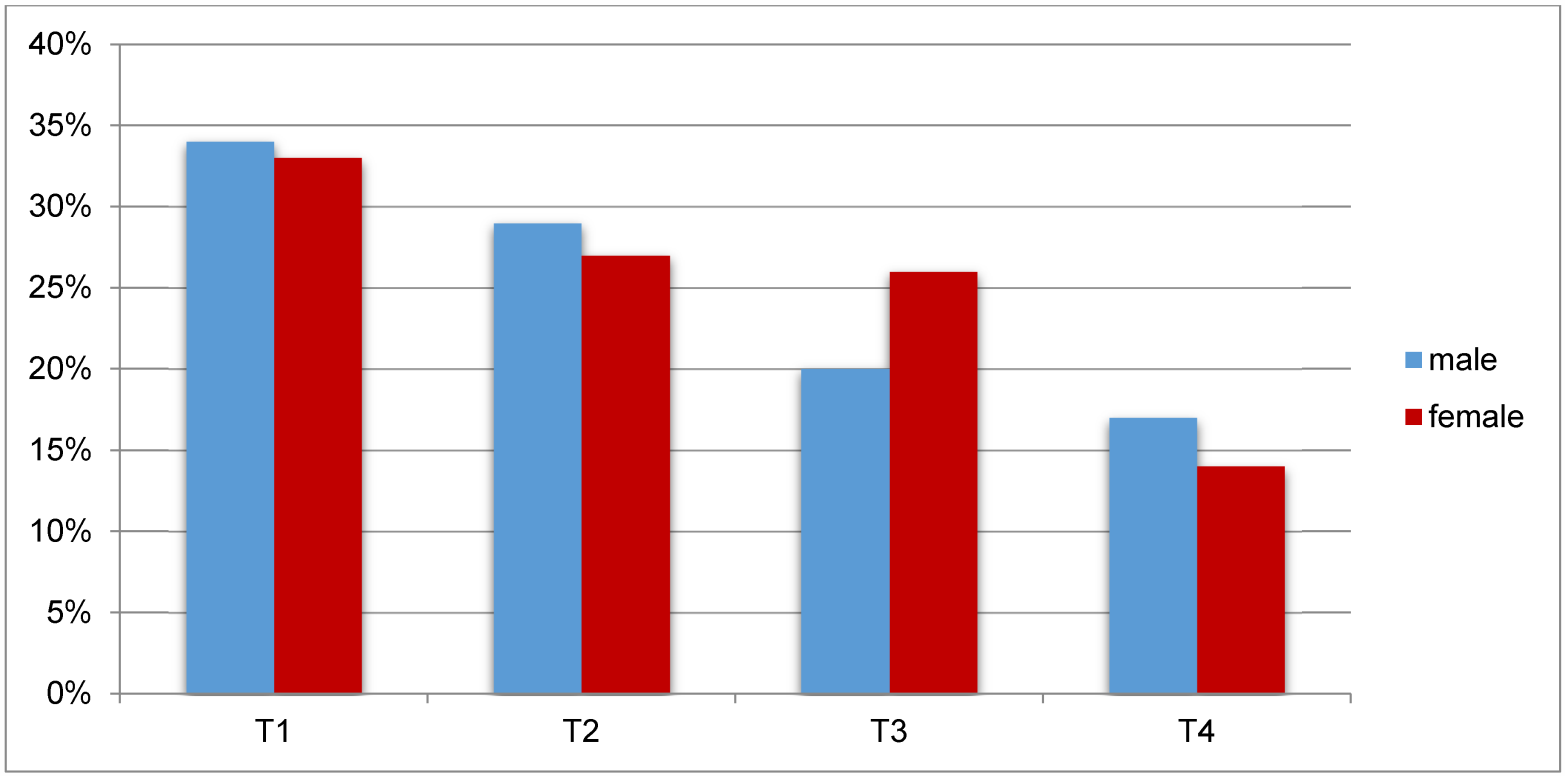
Distribution of T stages at the time of first diagnosis according to the gender in Germany in 2009/2010 [6]

In Germany, around 1/3 of all laryngeal cancer cases is diagnosed in an early stage (Figure 1 (Fig. 1)). The 5-year survival rate amounts to about 65% [6].

In Europe a declining incidence of laryngeal cancer is observed, especially for men [7]. In 2012, the age-standardized incidence of laryngeal cancer in European males amounted to 8.8 per 100,000; in the European Union it was 8.3 per 100,000 [8]. Based on the data of the World Health Organization, Chatenoud et al. [9] analyzed the mortality rate of laryngeal cancer during the last two decades in 34 European countries and the whole European Union (EU). While the mortality of male patients was nearly constant between 1980 and 1991, a decline of the mortality of 3.3% per year could be found from 1991 to 2012. From 1990 to 1991, the age-standardized mortality in the European Union amounted to 4.7/100,000; from 2000 to 2001 3.4/100,000; and from 2010 to 2011 2.5/100,000. The highest mortality rates (more than 6/100,000) were registered in Hungary, Moldova, and Rumania; the lowest (less than 1/100,000) in Norway, Iceland, and Sweden. With regard to females, a slight decline of the mortality of laryngeal cancer in the EU of 0.28/100,000 from 1990 to 1991, of 0.26/100,000 from 2000 to 2001, and of 0.23/100,000 from 2010 to 2011 was observed. 

**Figure 2:**
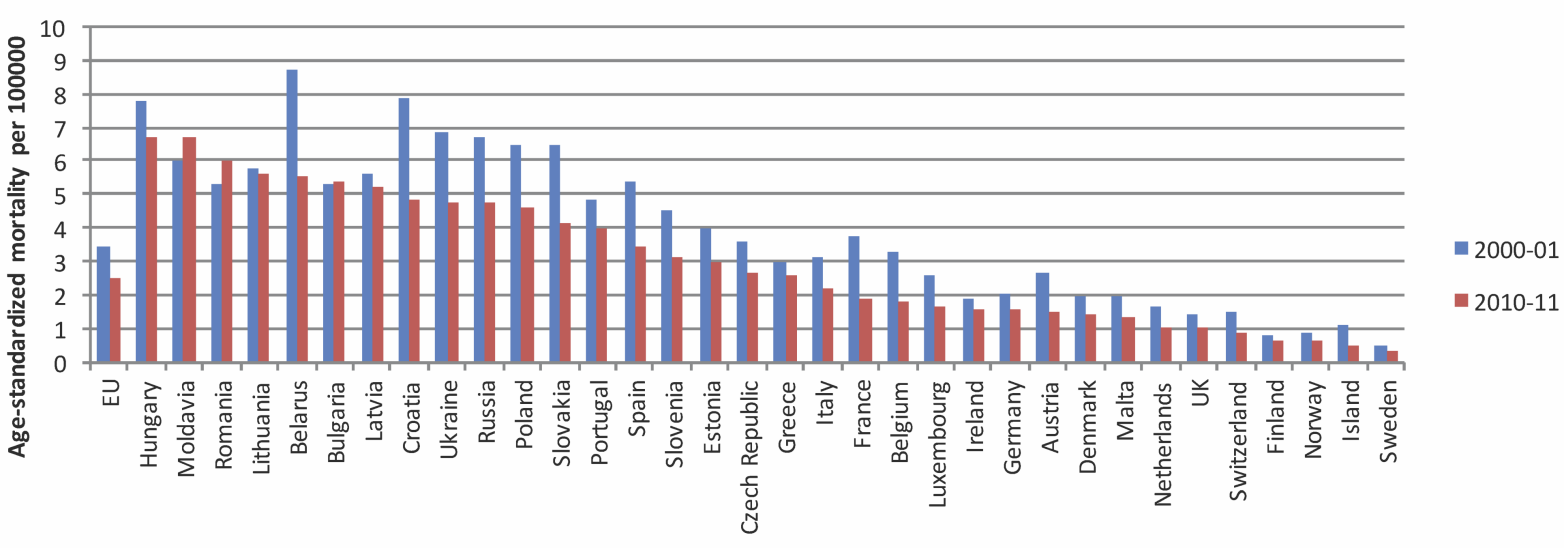
Age-standardized mortality of laryngeal cancer in male patients in selected European countries and the European Union in 2000/2001 and 2010/2011 [9]

**Figure 3:**
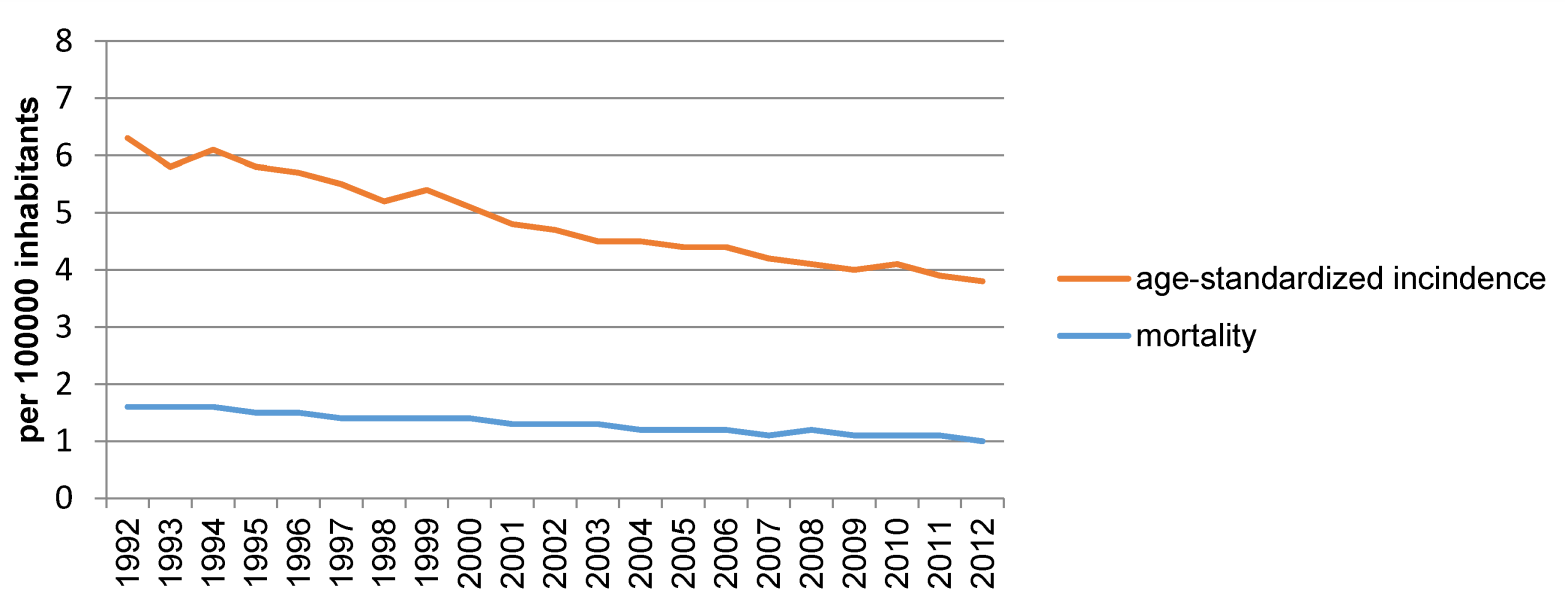
Age-standardized incidence and mortality of laryngeal cancer in the US from 1992 to 2012 [10]

**Figure 4:**
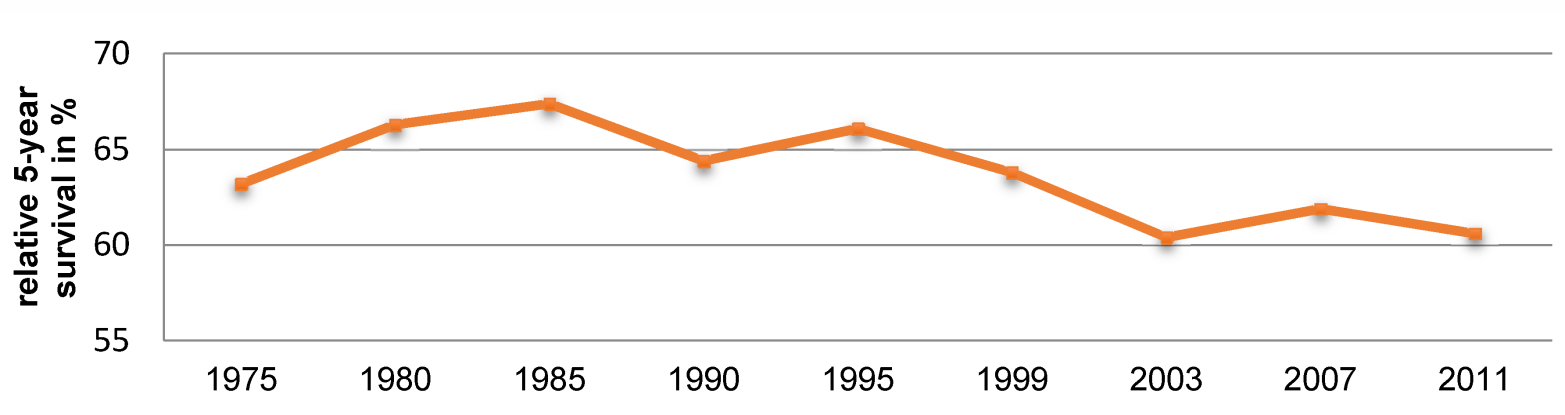
Relative 5-year survival after diagnosis of laryngeal cancer in the US from 1975 to 2011 [10]

The authors propose that the decline of the mortality rate is due to the reduction of tobacco consumption and, especially in Mediterranean countries, to the reduced consumption of alcohol (Figure 2 (Fig. 2)) [10]. However, it is relevant that not only the mortality but also the incidence of laryngeal cancer was declining during this period. Data from the US SEER (surveillance, epidemiology, end result) tumor registry confirm this observation [10]. This registry reveals a decline of the incidence of laryngeal cancer from 1992 to 2012 from 5/100,000 to 3/100,000 inhabitants (Figure 3 (Fig. 3)). At the same time, a decline of the mortality of laryngeal cancer was observed. However, there was no improvement of the relative 5-year survival rate in this period (Figure 4 (Fig. 4)) [10].

## 3 Therapy of primarily diagnosed laryngeal cancer

Surgical therapy modalities for laryngeal cancer encompass transoral surgery, open partial resection, and laryngectomy. Non-surgical curative treatment concepts include radiotherapy alone in cases of early stages of laryngeal cancer as well as combined radiochemotherapy and induction chemotherapy followed by radiotherapy. For most early laryngeal carcinomas, treatment with only one therapeutic modality (surgery or radiation) is sufficient. In cases of advanced tumors often multimodal therapy is required. The development of radiotherapy and the progress of transoral laser surgery reduced the impact of open surgery, especially regarding the treatment of early glottic carcinomas [11].

In contrast to surgical therapy, there are many data on radio(chemo)therapy of laryngeal cancer. Different radio(chemo)therapy protocols have been compared in numerous prospective randomized trials. Thus radio(chemo)therapy protocols are highly standardized, a randomized comparison to only surgical therapy is missing.

### 3.1 Evidence of transoral surgery of laryngeal cancer

#### 3.1.1 Significance of transoral laser surgery

Since the introduction of CO_2_ laser in laryngeal surgery by Strong and Jako [12], transoral laser surgery has gained in importance as resection technique with low morbidity as an alternative to external surgery especially in early cancer stages. The impact of laser surgery was elaborated in particular by groups from German speaking countries that could show excellent oncological outcomes after transoral laser surgery with lower morbidity in comparison to open partial resection [13], [14], [15].

##### 3.1.1.1 T1 and T2 carcinomas of the vocal folds

##### 3.1.1.1.1 Oncological results

Oncological data on transoral laser surgery of T1 and T2 carcinomas of the vocal folds are exclusively based on descriptive studies and meta-analyses (level III and IV evidence). Prospective randomized trials comparing the oncological outcomes of different therapeutic procedures are currently not available. 

Local control

The existing data on T1 glottic carcinomas reveal a good local control of 85–100% after transoral laser surgery, which is comparable to open partial laryngectomy (85–100%) and radiotherapy (84–95%) [16], [17], [18], [19], [20]. After transoral surgery of T2 glottic carcinomas, local control rates of 66-100% are described. Even in this context local control rates are similar to those of open partial resection (69–96%) and radiotherapy (50–85%) [19].

There are three meta-analyses [21], [22], [23] and one systematic review [24] comparing the local control after transoral laser therapy vs. radiotherapy of T1 and T2 carcinomas of the vocal folds. In summary, those four papers do not show a significant difference of the local control after transoral laser surgery and radiotherapy (Table 1 (Tab. 1)) [21], [22], [23], [24]. 

Overall survival

In the literature, the 5-year survival rate after laser surgical therapy of T1 carcinomas of the vocal folds amounts to 74–100% [25]. An analysis of 2,436 T1/T2 carcinomas of the vocal folds treated transorally, revealed a 5-year survival of 82% [26]. Regarding the overall survival, cohort studies on T1 laryngeal cancer (evidence level III) did not show any difference between surgical and radiotherapeutic procedures [27]. A systematic review from 2012 analyzing 17 studies could not identify significant differences regarding the overall survival between endolaryngeal surgery and radiotherapy [28]. 

Two meta-analyses are available that compare the overall survival of patients with T1 and T2 carcinomas of the vocal folds after transoral laser surgery and radiotherapy (Table 2 (Tab. 2)) [21], [23]. One of the studies revealed a significantly better overall survival after laser therapy of T1 and T2 carcinomas of the vocal folds [21]. The second one that assessed exclusively T1a carcinomas of the vocal folds also showed a tendency to a better overall survival after laser therapy; however, no significance could be confirmed [23]. Assessing the outcomes, it must be taken into consideration that the patients of the laser surgery group were selectively and not randomly assigned to the laser surgery group so that it seems to be possible that they had more superficial tumors in comparison to the patients of the radiotherapy group. Furthermore, because of the long inclusion period, a bias might be expected due to technological changes and changes of the treatment schemes. 

Disease-specific survival

In the literature, the 5-year disease-specific survival after transoral resection of T1 and T2 carcinomas of the vocal folds is reported to be 89–100% [25], [27], [29], [30], [31], [32], [33], [34], [35].

In a meta-analysis of 2011 on radiotherapy vs. laser therapy of T1a glottic carcinomas, 8 studies with an evidence level III and a total of 1,991 patients were evaluated regarding the disease-specific survival. No significant difference between both therapy concepts could be confirmed concerning the disease-specific survival, the odds ratio was 1.60 (95% confidence interval 0.79–3.26) in favor of laser surgery [23]. 

Larynx preservation and laryngectomy-free survival

Studies on larynx preservation after laser surgery of T1 and T2 glottic carcinomas report rates of 83–100% [36]. A systematic review of Yoo et al. [28] showed a tendency of better larynx preservation after initial transoral surgery compared to radiotherapy. A meta-analysis of Abdurehim et al. [23] on larynx preservation assessed data from 8 studies with 1,165 patients. Larynx preservation was significantly higher after transoral laser surgery than after radiotherapy. A meta-analysis of 606 patients with T1 and T2 glottic carcinomas summarized from 4 studies could not reveal a significant difference in the laryngectomy-free survival between radiotherapy and transoral laser surgery [21].

##### 3.1.1.1.2 Functional results

Voice quality

The voice quality after partial laryngectomy depends on the tissue volume which was resected in the area of the vocal folds but also on the aspect if other areas of the larynx might work as glottis substitute after surgery. Thus the voice quality may vary significantly despite similar interventions in different patients.

In the literature, the vocal quality of patients after transoral laser surgery is analyzed with subjective patient-based instruments and also with objective examiner-based methods. Using different measurements in different studies makes the comparison of the outcomes rather difficult. 

The vocal quality after transoral laser therapy and radiotherapy of T1aN0M0 glottic carcinomas was analyzed in a prospective randomized controlled trial from Finland [37]. The voice quality of 56 patients who were randomized in 2 therapy groups was compared. One group received transoral CO_2_ laser surgery, the other one radiotherapy of 66 Gy in daily fractions of 2 Gy for 6.5 weeks. The voice quality was assessed at the onset of study, 6, and 24 months after treatment based on the GRBAS scale (grade, roughness, breathiness, asthenia, strain), videostroboscopy, and self-evaluation of the patient. The assessment of the voice quality after therapy was similar in both groups, however, the voice of patients after transoral laser therapy showed more breathiness and patients often had an incomplete closure of the vocal folds. After 24 months, patients who had undergone radiation, reported less about impairment due to hoarseness in daily life. Within 2 years after randomization, 3 local recurrences occurred in both groups. The authors of the study reported about recruiting problems. They think that about 80% of eligible patients were not included in the study. The recruitment period of the study was 10 years. The authors conclude that primary radiotherapy is possibly the treatment of choice for patients with high requirements to an excellent voice quality [37].

Three systematic reviews of retrospective analyses investigated the voice quality after transoral laser therapy vs. radiotherapy of T1 and T2 carcinomas of the vocal folds. Spielmann et al. [38] analyzed the results of 15 studies on the voice quality after therapy of T1 and T2 glottic carcinomas. 12 studies did not reveal any difference of the voice quality, 3 studies reported about better results after radiotherapy. Feng et al. [22] investigated 293 patients with T1 and T2 glottic carcinomas from 6 studies with regard to their posttherapeutic voice handicap index (VHI). Three studies did not report any difference of the VHI between radiotherapy and laser therapy, in 2 studies the values after radiotherapy were significantly lower, in one study significantly higher. Because of the relevant heterogeneities of the studies, the authors could not perform a meta-analysis. In the most recent systematic review of T1 carcinomas of the vocal folds from 2012 published by Yoo et al. [28] radiotherapy was associated rather with measurable deterioration of the vocal quality but there were no significant differences in the patients’ perception. 

Currently, 3 meta-analyses of retrospective studies are available that compare the voice quality after transoral laser surgery with primary radiotherapy of T1 carcinomas [23], [39], [40], furthermore, there is one meta-analysis on T1 and T2 carcinomas [21] (Table 3 (Tab. 3)). None of the meta-analyses showed a significant difference of the voice quality between transoral laser therapy and radiotherapy. 3 meta-analyses showed a tendency to better vocal quality after radiotherapy [21], [23], [40], whereas 1 analysis revealed a tendency of better voice quality after laser therapy [39].

##### 3.1.1.1.3 Quality of life

Regarding the quality of life of patients with T1 and T2 glottic carcinomas, only level III evidence is found. Case control studies could not reveal a significant difference between the quality of life after laser therapy compared to radiotherapy [38]. A systematic review of 9 studies on quality of life after transoral laser therapy compared to radiotherapy could not detect a significant difference between both therapeutic modalities [38].

##### 3.1.1.1.4 Costs

On an international scale, it is not easily possible to compare costs of therapeutic procedures. The total costs of transoral laser surgery of T1 and T2 glottic carcinomas are lower than those of radiotherapy [22]. Higgins et al. [41] showed in a meta-analysis of 2011 that laser surgery is superior to radiotherapy regarding the cost-benefit ratio.

##### 3.1.1.1.5 Methodical annotations on systematic reviews and meta-analyses on T1 and T2 glottic carcinomas

Numerous systematic reviews and meta-analyses are available on the comparison of transoral laser therapy and primary radiotherapy of T1 and T2 glottic carcinomas. Those articles show that according to all primary analyses also the value of a systematic review or a meta-analysis depends on the subject investigated and which data were included. Depending on the parameters which should be evaluated, different inclusion and exclusion criteria could be defined so that the author of a review or a meta-analysis may influence the outcome of the analysis. Specifically considering different meta-analyses on the topic, it must be stated critically that none of the articles could identify and include a prospective, randomized controlled study. One reason for this is that planned randomized controlled studies have been interrupted or not even started because analyses on feasibility did not provide sufficient patient data [42], [43]. Hamilton et al. [44] analyzed responsible factors for the poor recruiting of the EaStER study (early stage glottic cancer: endoscopic excision or radiotherapy). They could show that the primary target parameter of the study was not accepted by all recruiting physicians and that partly the inclusion criteria were not applied. Furthermore, the physicians preferred the surgery arm which was also reflected in the passing on of study information to patients and made randomization difficult. Some centers complained about logistic problems of study participation [44].

The systematic reviews and meta-analyses varied with regard to defined target parameters and the type of included trials, so that based on the definition of the inclusion and exclusion criteria some studies were included in one meta-analysis and excluded in another one. Because of inherent bias and differences in the design of the included trials, meta-analyses of observational studies represent a particular challenge [45]. This aspect can be confirmed in this present review. The different target parameters and endpoints, as well as different measurement instruments, impede the comparability of study results and meta-analysis of data [46].

The advantage of Cochrane reviews is that they have to observe clearly defined, transparent, and supervised criteria for complete assessment, quality, and evaluation of published (mostly randomized) trials on a specific topic. A Cochrane review from 2014 that aimed at comparing endolaryngeal surgeries, open surgeries, and primary radiotherapy for treatment of early laryngeal cancer, identified only one single prospective, randomized controlled trial from 1990 that had compared open surgery and radiotherapy in 234 patients with T1 and T2 laryngeal cancer [47], [48]. No completed, randomized controlled trial analyzing endolaryngeal surgery could be identified [47].

##### 3.1.1.2 T3 and T4a glottic carcinomas

In case of appropriate patient selection, transoral surgery could be performed even in patients with advanced laryngeal carcinomas. The crucial aspect is a good exposition of the tumor. For transoral laser surgery of advanced glottic carcinomas, only level III and IV evidence is found. Already in 2001, Ambrosch et al. reported about 5-year survival rates of 68% and local control of 87% in a patient population of 70 patients with T3 carcinomas. Larynx preservation was possible in 85.7% of the patients [16]. A recent study confirmed the data in a larger population. In a retrospective trial of 226 patients with T3 glottic and supraglottic carcinomas who underwent transoral laser surgery, neck dissection (63%), and postoperative radiotherapy (18%), the 5-year survival rate amounted to 64.4% and the local control to 71.4%. Larynx preservation was possible in 87% of the cases [49]. 

Even carefully selected T4a laryngeal carcinomas may be resected transorally with laser surgery. Canis et al. [50] investigated the possibilities of transoral laser surgical resection of T4a laryngeal carcinomas. 79 patients with T4a supraglottic and glottic carcinomas were treated by transoral laser surgery with and without neck dissection. 39% of the patients received adjuvant radio(chemo)therapy. The local control after 5 years was 67.2%, larynx preservation was possible in 80% of the patients. The 5-year survival amounted to 65.3% in cases of glottic carcinomas and 49.9% in cases of supraglottic carcinomas. Canis et al. [50] came to the conclusion that transoral laser therapy with or without subsequent radiotherapy is a treatment option for selected patients with T4a squamous cell carcinomas of the larynx.

##### 3.1.1.3 Supraglottic carcinomas

Numerous studies are found on transoral laser therapy of supraglottic carcinomas. However, prospective randomized trials are missing. The evidence is based on retrospective analyses.

##### 3.1.1.3.1 Oncological results

Canis et al. [51] analyzed 277 patients after transoral surgery of supraglottic carcinomas, in 22% adjuvant radiotherapy was performed. After 5 years, control rates of 85% for T1 and T2 carcinomas could be achieved, 82% for T3 carcinomas, and 76% for T4 carcinomas. The 5-year disease-specific and the overall survival amounted to 92% and 76% in stage I and II, respectively; in stage III and IV it was 81% and 59%, respectively. Ambrosch et al. analyzed 48 patients with T1 and T2 supraglottic carcinomas who had a 5-year local control rate of 92% and 50 patients with T3 supraglottic carcinomas who had a local control rate of 86% [16]. In an analysis of 303 patients with T1 and T2 supraglottic carcinomas treated by transoral surgery, a 5-year survival of 70%, a disease-specific survival of 82%, and a local control of 90% was observed [26]. An analysis of transoral laser surgical interventions of T1-T4 supraglottic carcinomas revealed a local control after 2 years of 97%, the disease-specific 2-year survival rate was 80%, and the overall survival 85%. Larynx preservation was possible in 79% of the patients [52]. Iro et al. [53] analyzed 141 patients with T1-T4 supraglottic carcinomas who were treated with transoral laser surgery and if needed neck dissection and adjuvant radiotherapy. Local recurrences developed in 16% of the patients. The recurrence-free survival after 5 years was 65.7%, while this aspect depended on disease stage (stage I: 85.0%; stage II: 62.6%; stage III: 74.2%, and stage IV: 45.3%).

Motta et al. [54] reported about 5-year survival and local control rates after transoral laser therapy of T1 tumors of 91% and 82%, for T2 carcinomas of 88% and 63%, and for T3 tumors of 81% and 77%, respectively. Larynx preservation was possible in 87% of the T1 patients, in 85% of the T2 patients, and in 94% of the T3 patients [54]. In a study of 24 patients with T3 supraglottic carcinomas, Pantazis et al. [55] calculated a 5-year disease-specific survival of 91.7% as well as larynx preservation of 91.7%. Breda et al. [35] reported a larynx preservation of 90.7% in a study population of 43 patients with supraglottic carcinomas. An overview of the trials of the last 10 years regarding local control rates after laser surgical resection of supraglottic carcinomas is summarized in Table 4 (Tab. 4).

##### 3.1.1.3.2 Functional results

The functional results after transoral laser surgery of supraglottic carcinomas are good, permanent tracheostomy and functional laryngectomy as well as permanent PEG are rare [16], [53], [56], [57]. Ambrosch et al. [16] reported about 2% permanent tracheostomies in a population of 50 patients with T3 supraglottic carcinomas. In the investigations of Davis et al. [58] and Peretti et al. [59], permanent tracheostomy was necessary in none of the 46 and 20 patients, respectively, with supraglottic carcinomas. The need of permanent nutrition via stomach tube is given with 0–13% in studies on laser surgery of supraglottic carcinomas [16], [52], [58], [59]. Since there are currently no multicenter studies on functional outcomes after transoral laser surgery of supraglottic carcinomas, the results of the ongoing Supratol trial will be interesting. This trial aims at investigating if the good functional and oncological results after transoral surgery of supraglottic carcinomas may be confirmed in a larger prospective multicenter study of T2 and T3 supraglottic carcinomas of the larynx. 

#### 3.1.2 Significance of transoral robotic surgery

**Figure 5:**
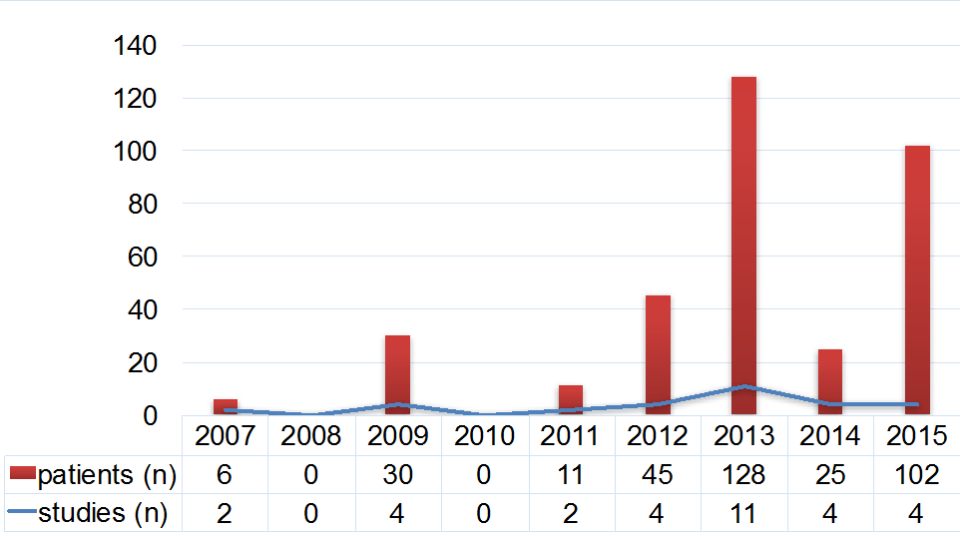
Number of studies and patients analyzed in studies on robotic surgery of laryngeal cancer from 2007 to 2014

Transoral robotic surgery (TORS) is the further development of transoral laser surgery. The worldwide mostly used system is the da Vinci^®^ robotic surgery system that is applied in ORs since 1998. The da Vinci^®^ system was developed by biomedical technicians of the US army in order to allow remote-controlled surgery in conflict areas. The first application in ENT was described in 2005 [60]. The surgeon controls the robot arms via a console. The instruments are inserted via the patient’s mouth and can be freely moved and controlled in a radius of 540°. The exposition of the larynx is performed via different mouth gags. Due to the use of different endoscopes and the included magnification function, a 3-dimensional visualization of the tumor is possible. The da Vinci^®^ system completes the existing procedures and enlarges the spectrum of possible interventions. The purchase price and costs for the instruments are a limiting factor of robotic surgery. Furthermore, the instruments of the da Vinci^®^ system for ENT surgery are rather big. Another inconvenience is the lack of an incorporated suction system so that an assistant is always necessary who is responsible for suction of smoke and blood in an already very narrow surgery site. Another restriction is the missing tactile and haptic feedback during surgery. The number of available trials on robotic surgery with the da Vinci^®^ system is summarized in Figure 5 (Fig. 5). Only level IV evidence is presently confirmed.

##### 3.1.2.1 Robot-assisted chordectomy

Only few case series are found on chordectomy with the da Vinci^®^ system. Kaythan et al. [61] treated 10 patients with T1 glottic carcinomas. The duration of surgery amounted to 21.6±6.75 minutes. In all patients negative resection margins were found. Lallemant et al. [62] also reported about successful chordectomy with negative resection margins, however, 2 cases developed recurrences in their study population. In case of concerns of applying the monopolar needle in the area of the vocal folds because of the broad coagulation zones, it is recommended to use CO_2_ fibers that cause less thermal damage [63], [64]. The already available studies do not show real advantages of robotic chordectomy in comparison to conventional laser microsurgery. Randomized studies are certainly the best instrument to elaborate the equivalence of transoral robotic and laser microsurgical chordectomy.

##### 3.1.2.2 Robot-assisted partial supraglottic resection

The most frequent robotic surgery of laryngeal carcinomas is partial supraglottic resection [65]. After the first report about the successful surgery of 3 patients by Weinstein et al. [66] some case series have been published that showed good oncological and functional results after robotic partial laryngectomy [67], [68], [69], [70], [71], [72]. The local control of those studies was between 80% and 100%, the overall survival rates are comparable to study data of open and laser microsurgeries. Only in rare cases tracheostomy or percutaneous gastrostomy were necessary. The duration of inpatient stay in those studies amounted to an average of 4 days [67], [68], [69], [70], [71], [72]. The authors consider the good overview of the surgery site as beneficial. It must be taken into account that the patients who underwent this type of surgery were a particularly selected group. So for example Ozer et al. [69] could only find 13 of 126 patients with supraglottic carcinomas who were eligible for robotic surgery. This selection bias must be taken into consideration when assessing the results.

Randomized studies comparing robotic partial supraglottic resection with open or laser microsurgery are currently not available. Ansarin et al. [72] compared retrospectively the first 10 patients who underwent transoral laser microsurgery in their institution from 2002 to 2005 with the first patients who underwent surgery with the da Vinci^®^ system from 2007 to 2011. In their patient population a shorter duration of surgery of robotic partial laryngectomy was observed while transoral laser microsurgery was superior with regard to better exposition of the larynx, free resection margins, and duration of necessary gastric tube. After a clearly shorter follow-up in the TORS group, no recurrences were observed in the TORS group, but 4 of 10 in the laser microsurgery group [72]. 

In summary, robotic partial laryngectomy seems to be a technically feasible option in selected patients. A recommendation with high grade of evidence, however, cannot be given yet because of the low numbers of patients included in the respective studies.

##### 3.1.2.3 Robotic total laryngectomy

The possibility to use the da Vinci^®^ system for robotic total laryngectomy was first described in 2013 [73]. After description of the surgical technique by Lawson et al. [73], 2 more case series have been published on robotic total laryngectomy [74], [75]. Robotic total laryngectomy seems to have two main advantages. First, pharyngotomy and the external skin incision are clearly smaller due to transoral preparation, which minimizes the risk of pharyngocutaneous fistula. Second, because of the missing lateral surgical preparation in contrast to open surgery, robotic laryngectomy allows preserving the fascia between neopharynx and carotid sheath that may serve as barrier and make rupture of the internal carotid artery less probable [65].

Surgeons consider robotic laryngectomy as technically safe procedure with good oncological and functional results. However, in their articles, the authors also describe that in 1 of 3 [74] and 2 of 7 [75] cases, respectively, conversion to open technique had to be performed. In addition, Smith et al. describe the occurrence of pharyngocutaneous fistulas in 2 of 7 cases, while the patient population consisted of patients with recurrent tumor disease and 6 patients underwent salvage surgery. Dowthwaite et al. [74] describe postoperative bleeding requiring revision.

The cases published up to now on robotic laryngectomy show that the patients have to be carefully selected and that robotic laryngectomy is not appropriate as standard procedure [74]. In the future, randomized studies should be conducted that evaluate robotic laryngectomy with regard to potential health-related benefit compared to regular laryngectomy. The significance of robotic laryngectomy cannot be finally assessed due to missing data. Since only few surgeries have been performed until today, this intervention has to be considered as experimental.

##### 3.1.2.4 Costs of robotic surgery

Beside the strict selection of the cases that may be treated with the da Vinci^®^ system and the missing availability of da Vinci^®^ robots in many hospitals, also the costs of da Vinci^®^ surgery must be considered in comparison to conventional surgery techniques. A cost evaluation in Belgium revealed average costs for open supraglottic partial resection (duration of surgery: 135–203 minutes) of 3,349 Euro, for transoral laser surgery (duration of surgery: 110–210 minutes) of 3,461 Euro, and for transoral robotic surgery (duration of surgery: 35–130 minutes) of 5,650 Euro. The total costs for laryngectomy amounted to an average of 3,581 Euro whereas robotic laryngectomy led to expenses of 6,767 Euro. Performing open and transoral laser surgery, the majority of the expenses (45%) were staff costs, the expenses of transoral robotic surgery were mainly (54%) due to equipment [76]. Even if those data cannot be transferred directly to the situation in Germany with its different remuneration system, this study shows the additional costs that arise due to robotic surgery of the larynx. Those increased expenses may only be justified if the benefit of robotic surgery can be proven in comparison to traditional procedures.

Since the technical feasibility of robotic surgery of the larynx by means of the da Vinci^®^ system could be shown in numerous studies, future evaluations have to assess the advantages and disadvantages of robotic surgery and to specify indications.

##### 3.1.2.5 Robotic surgery by means of new surgery robots

During the last years, other surgery robots have been developed especially for the narrow anatomical conditions in the area of the pharynx and larynx that will probably be the topic of future trials of the next years. Since the anatomy of many patients does not allow direct and rigid access, flexible systems were developed. The Flex^®^ system consists of a flexible endoscope, a 3D control console and a series of flexible instruments that were developed especially for the use in the neck area. The flexible endoscopy system disposing of 2 flexible working channels is controlled by the surgeon via a console. In contrast to the da Vinci^®^ system, the instruments give a tactile feedback. In a cadaver study, surgeries of the larynx could be performed successfully [77]. Meanwhile, first reports are available on the application of the Flex^®^ system for laryngeal surgery. The users consider the Flex^®^ system as promising option for surgery of the larynx, especially in cases of complex anatomy [78]. However, studies of larger patient populations are necessary in order to prove the reliability of the system. Currently there is no evidence to give a justified recommendation.

### 3.2 Evidence of open partial laryngectomy

Historically, open partial resections were mainly performed in Europe and South America. Since the introduction of transoral surgery of laryngeal cancer, the number of open surgical interventions has significantly decreased [11]. In former times, open partial laryngectomy represented a high percentage of interventions. Today the indication of open surgery of T1 and T2 tumors of the larynx is only rarely made in western industrial nations because retrospective case series showed similar oncologic results of open partial laryngectomy and transoral laser surgery for supraglottic and glottic carcinomas but an increased perioperative morbidity of open partial resection [57], [59], [79]. In particular classic vertical partial resections are more and more rarely applied. Open partial laryngectomy should be part of the repertoire of each head and neck surgeon in order to be able to consider it as therapeutic option. Open partial laryngectomy may is an option for patients with T3 tumors and selected T4 tumors as an alternative to primary radiochemotherapy or total laryngectomy. Another indication of open surgery is the treatment of T1 and T2 tumors if the larynx is difficult to expose, if transoral therapy is not possible or sufficient safety margins may not be kept, or if the anterior commissure is involved, which means a high recurrence rate after transoral laser microsurgery. The published articles on open partial laryngectomy cannot easily be compared and mostly correspond to level III evidence. Prospective randomized studies are currently not available.

One systematic review on open partial laryngectomy has been published. A total of 53 publications were eligible for inclusion and were assessed in the systematic review. The pooled local control rate of 5,061 patients after 24 months was 89.8%, the overall survival was 79.7% (n=3,967), and the disease-free survival was 84.8% (n=2,344). The perioperative mortality was 0.7%. 1.7% of the patients underwent laryngectomy because of functional reasons, decannulation was possible in 96.3% of the cases and permanent gastrostomy was required in 2% of the patients [80]. The meta-analysis moreover showed that no standardized measurement of the voice and swallowing function was performed in the included studies so that the functional outcome after surgery could not be adequately assessed [80].

The authors of the meta-analysis tried to summarize the results of primary studies on different open partial laryngectomies. So at a first glance, evidence seems to be found. On the one hand, more than 5,000 patients were included in the study; on the other hand, the analysis revealed a high heterogeneity between the single retrospective studies and it must be questioned if it is reasonable to evaluate different open partial laryngectomies in one analysis. The methodical flaws of the included articles that are also commented on by the authors of the meta-analysis, also evoke significant doubt if it can be considered as reasonable basis for evidence. 

A Cochrane review of 2014 intended to compare transoral surgery, open surgery, and primary radiotherapy, identified only 1 prospective randomized controlled trial from 1990 that compared open surgery and radiotherapy in 234 patients with T1/T2 laryngeal cancer [47], [48].

The 5-year survival rate of patients with T1 tumors amounted to 91.7% after radiotherapy and 100% after surgery, of patients with T2 tumors to 88.8% after radiotherapy and 97.4% after surgery. There were no significant differences of the survival rates of both groups. For patients with T1 carcinomas, the 5-year disease-free survival after radiotherapy was 71.1% and after surgery 100%; for T2 tumors it was 60.1% after radiotherapy and 78.7% after surgery. The difference of the 5-year disease-free survival of patients with T2 tumors was statistically significant [47]. Results regarding side effects, quality of life, voice quality after therapy, or costs could not be evaluated because of missing data [48]. Due to methodical flaws of the primary study such as imbalanced group sizes, insufficient pre-therapeutic staging, and poor follow-up, Warner et al. concluded that there is no sufficient proof to define the best therapeutic option [48].

#### 3.2.1 Significance of open vertical partial laryngectomy

Open vertical partial laryngectomies may be performed in cases of strictly unilateral laryngeal carcinomas, but also in selected tumors that have crossed the borders of one laryngeal side beyond the anterior commissure. Vertical partial resections are a therapeutic alternative for selected patients with T1 and T2 tumors for whom transoral laser surgery is not possible because of anatomical reasons. However, laser therapy is superior to vertical partial resection because of better postoperative voice and swallowing function and lower perioperative complication rates with comparable local control [81].

##### 3.2.1.1 Oncological outcome

The outcome of open vertical partial resection with regard to oncological safety is comparable to the one of transoral laser surgery. Large cohort studies could reveal good oncological results for vertical partial resections. Brumund et al. [82] reported about a patient population of 270 patients with T1–T3 glottic carcinomas having a 5-year survival rate of 83.1% for T1 tumors and 67.2% for T2 tumors. Perioperative complications occurred in 18% of the patients. The local control after 5 years was 91% for patients with T1 carcinomas. When the anterior commissure was not involved the 5-year-local control was even 96.2% and with involvement of the anterior commissure it was 74.7% for T1 tumors and 68.7% for T2 tumors. The larynx preservation was 93.3% [82]. Those data are confirmed by other retrospective trials reporting about local control rates of 87–100% for T1 and 68–88% for T2 tumors [83], [84], [85], [86], [87]. The clear difference of the local control rates between T1 and T2 tumors is certainly also due to the limited extent of resection of vertical partial resection. Studies from the last 10 years about the local control after open vertical partial laryngectomy are summarized in Table 5 (Tab. 5).

##### 3.2.1.2 Functional outcome

The voice quality after vertical partial laryngectomy depends on the location of the tumor and the performed surgery because it is decisive how much tissue was resected in the area of the vocal folds and the false vocal folds. On the other hand it is relevant if other areas of the larynx may replace the function of the glottis or if surgical reconstruction was performed. Singh et al. [88] could show that the voice quality after vertical partial laryngectomy is significantly different from healthy people and is similar to the one of patients after laryngectomy. Generally, a more or less obvious hoarseness is observed in all patients after vertical partial laryngectomy [89]. Biacabe et al. [90] could demonstrate that in 80% of the patients supraglottic structures are involved in laryngeal closure and phonation after fronto-lateral partial resection without reconstruction.

Despite the incomplete glottic closure, vertical partial laryngectomy is better compensated with regard to swallowing than horizontal partial laryngectomy [91]. The necessity of permanent gastrostomy is rare [89], [92]. Even permanent tracheostomy and functional laryngectomy are only needed in single cases [85].

#### 3.2.2 Significance of open horizontal partial laryngectomy

Among horizontal partial laryngectomies the partial resection according to Alonso as well as cricohyoido-epiglottopexy (CHEP) and cricohyoidopexy (CHP) must be mentioned [93]. Even regarding horizontal partial laryngectomy, no randomized controlled trials are available.

##### 3.2.2.1 Oncological outcome

In cases of supraglottic carcinoma, the supraglottic partial resection leads to excellent oncological results. Control rates of 90–100% for T1 and 80–97% for T2 carcinomas are described [36]. Survival rates of 75–80% for patients with stage III and 55–70% for patients with stage IV are reported [94]. An analysis of a larger patient population encompassing 407 patients described a local control rate of 86.5% after 5 years [95]. Another population of 267 patients with T1–T4 supraglottic carcinomas revealed a control rate of 92% after 5 years and a disease-specific survival of 73% [96]. Similar data are reported by Bron et al. [97] who evaluated patients with T1–T3 supraglottic carcinomas with a local control of 92.5% after 5 years. The overall survival was 75%. Organ preservation after supraglottic partial resection achieved values of 85% [94], however, in the literature the necessity of laryngectomy because of persisting aspiration is 3.5–12.5% [36].

Retrospective analyses report about 5-year local control of 84–95% after supracricoid partial resection [85], [98], [99], [100], [101], [102], [103], [104]. A patient population of 253 patients revealed survival rates after 3, 5, 10, and 16 years after supracricoid partial resection for glottic and supraglottic carcinomas of 85.8%, 79.1%, 57.6%, and 57.6%, respectively [97]. These data are confirmed by other studies that described 5-year survival rates of 66-88% [98], [99], [100], [101], [102], [103], [104]. Organ preservation is mostly possible. Laudatio et al. [102] reported about organ preservation in 97% after supracricoid partial resection in a cohort of 206 patients with T1–T4 carcinomas. Studies of the last 10 years on local control after open horizontal partial laryngectomy are summarized in Table 6 (Tab. 6).

##### 3.2.2.2 Functional outcome

The perioperative morbidity after open supraglottic partial resection is higher than after transoral laser surgery; the functional results with regard to swallowing and the necessity of permanent tracheostomy or secondary laryngectomy because of aspiration are poorer [105]. Prades et al. described pulmonary complications in 6% of the patients after supraglottic partial resection [106]. Sevilla et al. [96] reported about the necessity of permanent tracheostomy in 15% of the patients and functional laryngectomy because of aspiration in 9% of the patients. Usually, the voice, however, is not impaired after partial resection according to Alonso [107].

After supracricoid partial resection, however, a deterioration of the voice quality of different measure is reported [108]. Often the voice is breathy or rough, generally it improves after speech therapy. Regarding the incidence of postoperative swallowing disorders, different values are reported in the literature. Benito et al. reported about aspiration rates of about 30% after supracricoid partial resection, while 10% had severe aspiration [109]. In another investigation, persisting aspiration in 0.5–39% of the patients is described after supracricoid partial resection [102], [110], [111]. In cases of persisting aspiration without improvement despite therapy, the performance of functional laryngectomy must be considered. 

While some studies describe better functional outcomes after CHEP in comparison to CHP [112], [113], [114], other trials do not reveal any differences between both therapeutic procedures [115], [116], [117].

Swallowing disorders seem to be associated especially with the extent of the resection in the area of the epiglottis and the arytenoid cartilages [118]. Some studies show that a higher risk of aspiration is observed after arytenoid resection in the context of supracricoid partial resection [119], [109], [120], while other studies cannot confirm this correlation [112], [113], [118], [121], [122]. The risk of aspiration is clearly increased in patients who are older than 65 years [123], [124], [125] while higher ages of the patients are no contraindication for open partial resection with reasonable patient selection (good lung function, sufficient cognition and information about speech therapy) [126].

In a systematic review on swallowing disorders after supracricoid partial resection, Lips et al. [127] come to the conclusion that postoperative dysphagia clearly improves within 3 months after surgery and there are only low rates of severe complications after long-term follow-up. In 90-99% of the patients, the tracheostoma may be closed after supracricoid partial resection [107].

### 3.3 Evidence of total laryngectomy for therapy of laryngeal cancer

The first total laryngectomy was performed by Christian Albert Theodor Billroth in Vienna in 1873 [128]. His method did not include the opening of the pharyngeal tube, the hyoid bone and the epiglottis were preserved. The technique of total laryngectomy applied today was described by Bottini from Torino in 1875 [129]. Total laryngectomy could early achieve satisfactory oncological results in patients with laryngeal cancer. In 1991, a randomized trial could show for the first time that a non-surgical treatment concept (induction chemotherapy followed by radiotherapy) in patients with resectable laryngeal carcinoma did not lead to an impaired overall survival (after 2 years) in comparison to total laryngectomy and adjuvant radiotherapy [130]. After that, worldwide numerous randomized trials with the aim of laryngeal preservation were initiated for different radio(chemo)therapy protocols [131], [132]. The number of non-surgically treated patients with advanced laryngeal cancer increased during the last decades due to optimized radio(chemo)therapy protocols. Between 1997 and 2008, the number of total laryngectomies was reduced by 48% in the US, at the same time, the number of newly diagnosed laryngeal cancer decreased by 33% [133]. A study with 5,394 patients with T3 and T4 laryngeal cancer of the SEER tumor registry revealed an increase of the percentage of non-surgically treated patients from 32% to 62% from 1992 to 2009 [134]. An evaluation of 14,000 patients with laryngeal cancer of the Dutch cancer registry confirmed this tendency [135]. The mortality after total laryngectomy amounted to 1.4% in the US in 1997 and 1.1% in 2008 [133].

#### 3.3.1 Oncological results

For patients after total laryngectomy or adjuvant radio(chemo)therapy of T3 tumors, local control rates of 69–87% and 5-year survival rates of 53-86% were reported [136], [137], [138]. Already in 1991, it could be shown that patients with T4a carcinomas benefit from total laryngectomy because they respond more badly to a primarily non-surgical treatment concept and have a poorer survival rate than patients who underwent primary surgery [130]. This aspect was meanwhile confirmed by numerous retrospective studies. Karatzanis et al. [139] analyzed 384 patients who were treated between 1980 and 2007 for T4a laryngeal cancer. In the group of primarily surgically treated patients a disease-specific 5-year survival of 62.2% was found while in the group that was treated with primary radio(chemo)therapy the disease-specific survival amounted to 24.5% and the overall survival after 5 years was 16.7%. Those differences were statistically significant [139]. In a study of Gourin et al. [140] of 451 patients with laryngeal carcinomas who were treated between 1985 and 2002, patients with T4 carcinomas had a significantly better survival after surgery (55%) in comparison to radiochemotherapy (25%) or radiotherapy alone (0%). Furthermore, the authors could show that functional impairment of the larynx before therapy generally persisted until after the end of radio(chemo)therapy [140]. A recent retrospective analysis of 969 patients with T4a laryngeal cancer performed by the National Cancer Database confirmed that total laryngectomy ± adjuvant therapy was superior to organ-preserving therapy with regard to survival. The survival after total laryngectomy with or without adjuvant therapy was evaluated in comparison to the survival after primary radiochemotherapy. 616 patients received primary radiochemotherapy, 353 underwent primary total laryngectomy. The median survival was significantly different between the different therapeutic options. After primary total laryngectomy it was significantly longer and amounted to 61 months whereas the median survival after primary radiochemotherapy was 39 months. Additionally, it was revealed that patients who were treated in high-volume hospitals underwent more frequently total laryngectomy [141]. This aspect corresponds to a better patient selection in specialized centers.

A retrospective study of 5,394 patients with T3 and T4 laryngeal carcinomas who were treated between 1992 and 2009 showed that patients who had received surgical therapy had better 2-year and 5-year disease-specific survival rates (70% vs. 64% and 55% vs. 51%, respectively, p<0.001) and 2-year and 5-year overall survival rates (64% vs. 57% and 44% vs. 39%, respectively, p<0.001) compared to patients who had received non-surgical therapy [134].

In an analysis of 3,794 patients who were treated in the Netherlands between 1991 and 2010, a 5-year overall survival of 49% was revealed for 2,072 T3 laryngeal carcinomas after total laryngectomy ± adjuvant radiotherapy, 47% after primary radiotherapy, and 43% after primary radiochemotherapy. Patients with T4 laryngeal cancer revealed a significant difference of the 5-year survival rate. After total laryngectomy ± adjuvant radiotherapy it amounted to 48%, after primary radiotherapy to 34%, and after primary radiochemotherapy to 42% [135]. An evaluation of 221 patients with T4 carcinomas performed by Rosenthal et al. [142] revealed a significantly better local control after total laryngectomy and postoperative radiotherapy in comparison to larynx preservation protocols. 

In a systematic review of 24 studies on the overall survival of patients with T4a laryngeal carcinomas, the overall survival after 2 years after radiation amounted to 12–21.2%, after radiochemotherapy to 30–65%, and after surgery to 30–100%. After 5 years, the overall survival was 0–75% after radiotherapy, 16–50.4% after radiochemotherapy, and 10–80.9% after surgery [143]. These data show that total laryngectomy is associated with a high survival rate of patients with T4a laryngeal cancer. 

Assessing those retrospective studies, it must be taken into consideration that the radiotherapy protocols have been modified during the last decades and chemotherapy was added to the treatment protocols. The necessity to perform prospective randomized trials that meet the methodical parameters of evidence-based medicine becomes obvious.

In summary, it can be stated that total laryngectomy is an oncologically safe procedure for therapy of advanced laryngeal cancer and tumor recurrences even if the number of total laryngectomies decreased due to optimized radiochemotherapy and increasing performance of minimally invasive surgical procedures. According to the current state of knowledge, especially in cases of broad infiltration of the thyroid cartilage, total laryngectomy should be preferred because of oncological and functional aspects. Also in cases of advanced functional impairment of the larynx before therapy, total laryngectomy should be performed. 

#### 3.3.2 Functional results

Voice rehabilitation after total laryngectomy is possible by means of esophageal speech, tracheoesophageal speech with voice prosthesis, and electrolarynx. In a systematic review on voice rehabilitation after total laryngectomy, it became obvious that the success rate of esophageal speech is poorer than the one of electrolarynx and tracheoesophageal speech with voice prosthesis [144]. Patients using tracheoesophageal voice prostheses report about significantly better voice quality than with electrolarynx or esophageal speech [144], [145]. A systematic review about the outcome after pharyngolaryngectomy reports about the same result [146]. Also patient satisfaction and quality of life are best in patients who received voice prostheses [144]. Some authors consider tracheoesophageal voice prosthesis as gold standard of voice rehabilitation [147]. One disadvantage of the voice prosthesis is the regular change of the prothesis and the high number of shunt insufficiencies that is characterized by transition of liquid from the esophagus into the trachea along the inserted voice prosthesis. A systematic review by Hutcheson et al. [148] described an incidence of shunt insufficiencies of up to 29%. In summary, however, many patients are not satisfied with their voice and ability of communication after total laryngectomy [149].

Patients after total laryngectomy alone have a better vocal function than patients after total laryngectomy with adjuvant radiation or salvage laryngectomy [145].

Dysphagia in patients after total laryngectomy is a frequent phenomenon [150]. The incidence of dysphagia as consequence of the altered swallowing mechanism after total laryngectomy is given with 10–58% [151], [152].

Total laryngectomy and adjuvant radiotherapy is superior to organ-preserving therapy in cases of T4 carcinomas with regard to late functionality [142]. Patients who received primary radiochemotherapy for organ preservation have significantly higher dysphagia rates compared to laryngectomees [142], [149]. Furthermore, patients after total laryngectomy alone have better swallowing function than patients after total laryngectomy and adjuvant radiation or salvage laryngectomy [145], [153], [154].

#### 3.3.3 Quality of life

The loss of the larynx means an important turning point for patients having undergone total laryngectomy. A German prospective multicenter study could show that the quality of life after total laryngectomy first becomes poorer, but in some areas (global health status, cough, weight) recovers during the first year after surgery, while other fields (physical functionality, role function, social function, sleepiness, stridor, loss of appetite, financial difficulties, senses, speech, and social contacts) could not achieve the preoperative level [155]. Similar data are confirmed by other studies. Villaseca et al. [156] could not reveal striking features regarding the global health status of patients at least 2 years after total laryngectomy, but a reduced physical functionality compared to healthy people. In an analysis on the quality of life published by Perry et al. [157], also a significantly reduced physical health, reduction of the social relations, and a higher susceptibility to depression and anxiety after total laryngectomy were obvious in comparison to healthy people. One year after total laryngectomy, more than one third of the patients only participate rarely in social life [158] and only very few laryngectomees of working age succeed in professional re-integration [159]. The psychic well-being after total laryngectomy is severely limited [157]. Also partners of laryngectomees suffer from the enormous psychic stress and need psycho-oncological care [160].

Some studies have been published that show that the quality of life after total laryngectomy and after primary radiochemotherapy is comparable [161], [162]. Other studies reveal a better quality of life after non-surgical therapy in comparison to total laryngectomy [163], [164], [165].

#### 3.3.4 Costs

In comparison to non-surgical therapeutic procedures, the costs of total laryngectomy can only hardly be compared on an international scale because of the different healthcare systems. Specific data are available for the US. An analysis of the SEER tumor registry with evaluation of 5,038 cases in 1997 and 3,414 cases in 2008 revealed mean hospital costs for laryngectomies of $ 58,000 in 1997 and $ 109,000 in 2008, which – according to the authors – is congruent to the overall development of the healthcare costs in the US [133]. Additionally, an increase of the mean duration of inpatient stays from 13 to 14 days was observed. As possible explanation, the authors mention an increasing number of salvage surgeries and higher complications rates in the context of overall less performed interventions. In a retrospective study, Dedhia et al. [166] demonstrated that the surgery costs accounted for an average of 24% of the whole hospital expenses while rooms, respiration therapy, laboratory, pharmacy, and radiology made up 38%, 14%, 8%, 7%, and 3%, respectively. In a model of decision analysis with data from studies, case series, and meta-analyses, Davis et al. [167] came to the conclusion that total laryngectomy with postoperative radiotherapy costs nearly $ 3,000 less than induction chemotherapy followed by radiotherapy.

#### 3.3.5 Use of stapler during total laryngectomy

The use of stapler for pharyngeal closure during total laryngectomy was first described in 1973 [168] and since then it was investigated in numerous retrospective trials. The main advantage of the stapler is the substantial intraoperative time saving. The main disadvantage of this technique is that the tumor is not visualized during resection so that oncological risks may occur, especially when the cases were not carefully selected before surgery. It is recommended to use the stapler only for endolaryngeal tumors [169]. In comparison to manual closure, closure with the stapler depends probably less on the capabilities of the surgeon so that it can be assumed that the successful pharyngeal closure mainly depends on tissue parameters and less on manual skills of the single surgeon. If surgeons know how to use the stapler, individual factors would be nearly eliminated so that Bedrin et al. called the stapler “the great equalizer” [170]. However, similar to manual closure of the pharynx, there is no standardized application which makes comparison of the results of single studies rather difficult.

On the one hand, there are differences regarding the number of staple lines used for pharyngeal closure. Some surgeons consider a single line as sufficient [171], whereas others prefer double lines [170], [172]. The pre-laryngeal muscles may also be sutured in order to enforce the pharyngeal closure [171], many authors, however, think that this is not necessary [170], [173]. Additionally, staplers of different manufacturers and different lengths are applied [171], [174].

Some prospective, non-randomized studies are available that show a low rate of pharyngo-cutaneous fistulas in small patient populations after stapler in comparison to manual pharyngeal closure [175], [176]. The rate of fistulas, however, were very high in both patient groups after manual closure with 19.8% and 36.7% [175], [176].

The largest retrospective investigation with 1,415 patients who underwent pharyngeal closure by means of stapler during total laryngectomy, showed an overall incidence of pharyngo-cutaneous fistulas of 11.9%, while the rate of fistulas amounted to 19.4% in patients who had received primary radiotherapy with 60–65 Gy. Patients after incomplete radiation (40–45 Gy) had a fistula rate of 9.2%, and non-radiated patients of 5%. The authors reported no increase of the recurrence rate in comparison to conventional technique and lower costs due to time saving [170].

In a systematic review on the effectivity of stapler in comparison to suture of the pharynx during total laryngectomy, only 4 studies with 417 patients could be included [177]. In the stapler group, the incidence of pharyngo-cutaneous fistulas was 8.7%, while it amounted to 22.9% in the suture group, corresponding to an absolute risk reduction of 15%. The duration of surgery in the stapler group was shorter of an average of 80 minutes, the inpatient stay could be reduced of an average of 6 days, but only 2 of 4 included studies contained data on the durations of surgery or the time of hospitalization.

Interpreting those results, it must be taken into account that a selection bias exists because in the group that had manual closure by means of pharyngeal suture mostly patients with hypopharyngeal, oropharyngeal, or extralaryngeal tumors were found, while in the stapler group only patients with endolaryngeal tumors were treated. Patients with tumors extending into the hypopharynx or oropharynx or with extralaryngeal tumor growth have a higher risk to develop pharyngo-cutaneous fistulas because of the generally larger resection wound at the pharynx. The authors of the systematic review describe further methodical flaws of primary studies such as for example the missing definition of patient selection criteria or missing clinical data. In the sense of evidence-based medicine, the results of this review article are not sufficiently valid because the evidence levels of the different studies are rather low. This conclusion is not due to the performance of the review article but – as described by the authors in the systematic review – to the poor quality of the available literature [177].

#### 3.3.6 Significance of including the thyroid gland in the laryngectomy specimen

Because of the neighborhood of the thyroid gland to the laryngeal skeleton, infiltration of the thyroid gland by laryngeal carcinomas is possible. Tumors of the larynx can infiltrate the thyroid gland due to continuous growth or lymphogenic metastasis [178], [179], [180]. Up to now, only one case report has been published regarding hematogenic metastatic spread into the thyroid gland diagnosed in a patient with hypopharyngeal cancer [181].

Different anatomical weaknesses were identified in the laryngeal skeleton that allow infiltration into the thyroid gland. In the subglottic space, the ligamentum cricothyroideum and the area located laterally are considered as crucial in this context [182], [183]. Additionally, the paraglottic space between the mucosal surface of the larynx and the cartilage below is considered as relevant landmark for extralaryngeal growth. No fascia are found so that organ-crossing growth is possible and tumor expansion can occur especially into antero-inferior direction [178]. Because of the extensive network of lymphatic and blood vessels in the thyroid gland, tumor spread within the gland is possible and multifocal growth is often observed [178], [183]. The presence of infiltration of the thyroid gland is probably overestimated by preoperative imaging [184]. 

Two meta-analyses from 2009 [185] and 2013 [186] analyzed risk factors for infiltration of the thyroid gland by squamous cell carcinomas of the larynx. A meta-analyses published by Mendelson et al. [185] included 8 studies with 399 patients. Tumor infiltration of the thyroid gland was found in 8% of the patients, mostly occurring by continuous growth. Subglottic expansion of more than 10 mm, transglottic tumor growth, and subglottic tumors were associated with infiltration of the thyroid gland. Surprisingly, the presence of cartilage infiltration by the tumor was no significant predictor for infiltration of the thyroid gland, however, the significance was limited by the fact that only 2 relatively small studies had performed such an analysis [185]. The meta-analysis performed by Kumar et al. [186] included 16 studies. The incidence of infiltration of the thyroid gland amounted to 10.7% of a population of 1,287 patients. Patients with primarily subglottic tumors and tumor spread into the subglottis had a clearly higher risk of thyroid infiltration [186].

Patients with diagnosed infiltration of the thyroid gland by laryngeal carcinoma have a poorer prognosis and often die of recurrent tumors [183], [187]. Probably, this is less frequently caused by infiltration of the thyroid gland itself but rather by the characteristics of the tumors infiltrating the thyroid gland: advanced transglottic tumors with infiltration of the subglottis and primarily subglottic tumors [185], [186]. Further studies are required to assess the actual influence of an infiltration of the thyroid gland on the survival.

Currently there is no evidence regarding the extent of thyroid resection during total laryngectomy. Mendelson et al. [185] recommend ipsilateral lobectomy and resection of the isthmus. Because of the lack of data, the inclusion of (parts of) the thyroid gland into the laryngectomy specimen cannot be recommended as routine procedure for each total laryngectomy. In patients with infiltration of the thyroid gland confirmed by imaging or with the risk profile defined in meta-analyses, i.e. transglottic tumors, subglottic tumors, and tumors with subglottic growth of more than 10 mm, the inclusion of (parts of) the thyroid gland into the laryngectomy specimen should be taken into account. Also for this issue, no evidence of level I exists.

## 4 Therapy of recurrences after primary radio(chemo)therapy

Residual tumors and recurrent tumors of the larynx after radiotherapy represent a severe clinical challenge. They are mostly characterized by aggressive growth, unpredictable lymphatic flow, and they are associated with a poor local control. The time up to the development of recurrence does not only depend on the tumor biology, but also on different parameters of primary therapy such as for example the radiation dose. The majority of recurrences occurs within the first 2 years after primary treatment; recurrences developing within the first 10 months after primary treatment are associated with a particularly poor prognosis [188]. Curative treatment options for recurrences and residual tumors after primary radio(chemo)therapy are total laryngectomy, open partial laryngectomy and transoral (laser) surgery, while worldwide total laryngectomy is most frequently performed in cases of recurrences [189], [190]. The reason is that total laryngectomy is technically easy to perform and the outcome is well investigated. However, total laryngectomy is associated with severe consequences with regard to functionality and quality of life.

Many patients with residual tumors or recurrences after failed primary radiochemotherapy suffer from advanced tumors and nearly half of the patients have transglottic T3 or T4 tumors [191]. In such situations, imaging and endoscopy often lead to misinterpretation of the tumor size in the sense of “understaging” because the assessment of the tissue is very difficult due to post-radiogenic edema and scarring. In cases of recurrences, pre-therapeutic clinical examination and imaging only have an exactness of about 50%, while up to 90% of the recurrent tumors are estimated too small [192]. Furthermore, often multicentcric tumor foci are found in cases of recurrent tumors that may grow beyond the size of the primary tumor [192]. A recent retrospective analysis of 173 patients with tumor recurrences after radiotherapy of early glottic carcinomas revealed that 61% of the recurrences had a higher rT stage, 31% had the same rT stage, and only 8% had a lower rT stage in the sense of down-staging in comparison to the initial T stage of the primary tumor [193]. That is why in recurrences after primary radio(chemo)therapy generally resection at least in the limits of the original tumor is appropriate. Shah et al. [194] recommend partial resection in T1 and T2 recurrences only when the recurrent tumor does not exceed the original tumor extension.

Successful management of recurrences depends on strict selection criteria. Especially when planning transoral surgery and open partial laryngectomy, careful assessment of the location, size, and extension of the tumor recurrence should be performed. R0 resection is prognostically crucial in recurrences and should be the first objective of surgery [188], [194]. R0 resection is complicated by post-radiogenic changes of the tissue that also makes preoperative estimation of the tumor size difficult. Thus, intraoperative section analyses should be performed to ensure tumor-free resection margins.

### 4.1 Significance of transoral surgery after radio(chemo)therapy

There are numerous retrospective studies on transoral laser surgery in selected patients with circumscribed recurrent tumors after radiotherapy of early laryngeal carcinomas. They showed acceptable oncological results and healing with organ preservation after single or multiple transoral laser therapy may be achieved. Prospective trials investigating the outcome after surgery in recurrences after primary radio- or radiochemotherapy are rare [195]. The evidence is mainly based on retrospective case series [196], [197], [198]. The disease-free survival after transoral laser therapy of recurrences after primary radio(chemo)therapy amounts to 43–90% [199], [200]. In the literature, the local control after single transoral laser therapy of tumor recurrences varies between 38% (in this study also advanced tumors had been treated) [201] and 85% [195]. Repeated laser surgical resections of re-recurrences after previous radiotherapy are possible and may improve the local control as shown by Steiner et al. who could increase the local control from initially 38 to 71% by repeated laser surgical resection [179]. Other authors report about local control rates of 50–82% after multiple laser resections carried out after primary radio(chemo)therapy [202], [203]. On the other hand, Roedel et al. recommend to consider early salvage laryngectomy in cases of re-recurrences after transoral laser surgery because the local control after repeated transoral surgery seems to be a problem [196].

A limiting factor of these results is that transoral laser surgery in cases of recurrences after previous radio(chemo)therapy can only be performed in selected patients with circumscribed recurrences in the mobile soft tissue of an irradiated larynx. Quer et al. [197] consider vocal fold paresis, subglottic extension of more than 5 mm, and infiltration of the thyroid cartilage as contraindication of laser therapy in recurrences.

In 2014, a meta-analysis on transoral laser surgery after primary radio(chemo)therapy was published. 11 studies were included in this meta-analysis while 9 publications originated from Europe and 2 from the US [198]. Only one of the included studies was prospective. The 2-year overall survival after transoral laser surgery in recurrences amounted to 74.8%. Larynx preservation was possible in 72.3% of the patients. The local control of 286 patients was 56.9% after one laser surgical intervention and 63.8% after multiple laser surgical interventions, and 88.2% after salvage laryngectomy [198].

For all retrospective studies on transoral surgery, it must be taken into account that selection bias is present because a good exposition with the rigid tube is a precondition for transoral laser surgery. Thus the results are difficult to compare with the results of other procedures. Furthermore, in this meta-analysis nearly exclusively patients with T1 and T2 tumors had been treated so that a very selected patient population is found. It is alarming that no statement on the voice and swallowing function after surgery of recurrences could be made in this meta-analysis because the data of the primary studies were not sufficient.

Taking the existing evidence into account, the conclusion may be drawn that transoral laser surgery in recurrences after previous radiation should be performed only in carefully selected cases and in centers having a high expertise in transoral laser surgery. 

### 4.2 Significance of open partial laryngectomy after radio(chemo)therapy

Some publications are available on the safety of open partial laryngectomy in cases of residual tumors or recurrences after primary radiochemotherapy with regard to local tumor control, overall survival, and postoperative voice and swallowing function. However, the performance of open partial resection in recurrences is not broadly performed and in many countries it is not applied [204]. Most publications on open partial laryngectomy are from the same few centers and only refer to small patient populations.

The early diagnosis of recurrences is a key factor in order to possibly perform larynx preserving surgery. Also in this context, the patient selection is crucial. Patients with arytenoid fixation, tumor involvement of the interarytenoid region, infiltration of the pre-epiglottic space, subglottic involvement, or extralaryngeal growth should undergo total laryngectomy [205]. Considering those exclusion criteria, oncologically safe partial laryngectomy is possible in selected patients. However, patients who are eligible for partial laryngectomy have to have good pulmonary function and few comorbidities and furthermore the compliance to accept a possibly long swallowing rehabilitation.

In most of the studies supracricoid partial resection was carried out. The local control was 57% [206] to 100% [207], [123], [208], [209], [210], [211], [212] in retrospective studies, while each of those studies encompassed patient populations of less than 16 patients. One study of 78 patients who received supracricoid partial resection for tumor recurrence after primary radiotherapy revealed a local control rate of 95% [213]. According to the literature, the 2-year disease-free survival is between 73% [214] and 100% [123], [211], [212] and the overall survival after 2 years is between 71% [215] and 100% [216].

In the studies on open partial laryngectomy for recurrences after radio(chemo)therapy, the postoperative quality of the voice and swallowing is only rarely assessed and not systematically analyzed [204]. Most authors describe satisfactory postoperative speech results in 76–87% of the patients [205], [217]. The studies depict different protocols on swallowing rehabilitation and transition to normal diet which makes comparison difficult. Even if some authors promote prophylactic insertion of PEG tubes [123], most of the authors recommend postoperative nutrition by means of nasogastric tubes. Permanently necessary nutrition via PEG tube is reported only in very few studies [123], [218], [219]. The incidences of postoperative dysphagia are mostly described as low, often those data are inexact and not systematically evaluated and quantified [204], [205].

One meta-analysis of retrospective trials on this topic has been published. Paleri et al. [204] could show in this meta-analysis of 26 publications on different open partial laryngectomies after primary radio(chemo)therapy an overall survival of 83.1% in a population of 560 patients. Larynx preservation was possible in 83.9% of the cases. The local control rate amounted to 86.9%. Decannulation was possible in 95.1% of the cases who had undergone partial laryngectomy. The limiting factor of the results of this study is that nearly exclusively patients with T1 and T2 tumors had been treated and different open partial laryngectomies had been evaluated. Meta-analysis of the postoperative quality of swallowing or voice could not be performed because the data of the primary studies were not sufficient [204].

Comparing surgical treatment alternatives, the oncological results of transoral laser surgery are poorer than those of open procedures as described by Paleri et al. [204]. This is also applicable when the improved local control after repeated laser surgical interventions is taken into account [198].

### 4.3 Significance of total laryngectomy after radio(chemo)therapy

Salvage laryngectomy after primary radio(chemo)therapy is often the only, possible curative therapeutic option for patients with advanced laryngeal cancer. It is an integral part of multimodal therapy of advanced laryngeal carcinomas. In the prospective randomized RTOG 91-11 study salvage laryngectomy became necessary in 129 of 517 patients after initially larynx-preserving therapy. The incidences amounted to 28% after chemotherapy followed by radiotherapy, 16% after concomitant radiochemotherapy, and 31% after radiotherapy alone. The occurrence of complications after salvage laryngectomy ranged between 51% and 59%, while no significant difference between the groups was observed. The 2-year overall survival rate was 69% after chemotherapy followed by radiotherapy, 71% after concomitant radiochemotherapy, and 76% after radiotherapy alone. In summary, 5% of the patients had to undergo total laryngectomy because of functional reasons, e.g. because of aspiration [190].

Salvage laryngectomy is characterized by complicated intraoperative dissection because of scarring and insufficient blood supply of the tissue after radiotherapy. There is a higher postoperative complication rate that is mainly determined by impaired wound healing, laryngeal edema, and the development of salivary fistulas. Pharyngocutaneous fistulas are the most frequently occurring surgical complication in the postoperative phase after total laryngectomy, especially in salvage situations. In a study of Weber et al., the incidence of pharyngocutaneous fistulas was highest after concomitant radiochemotherapy (30%) and lowest after radiotherapy alone (15%) [190].

Pharyngocutaneous fistulas are associated with increased morbidity, prolonged hospitalization, and higher costs and predispose for vascular arrosion [220]. The incidence after total laryngectomy varies in different studies between 3% and 65% [177]. In 3 meta-analyses the main risk factors for the occurrence of pharyngocutaneous fistulas were analyzed [221], [222], [223]. From a methodical point of view, it must be criticized that most of the studies included in the meta-analyses had only low case numbers and in most cases no multivariate statistical analyses were performed. All 3 meta-analyses concur that the condition after preoperative radiotherapy and the presence of a pre- or postoperative hemoglobin value <12.5 g/l is associated with an increased risk to develop pharyngocutaneous fistulas [221], [222], [223]. Additionally, at least one of the meta-analyses could show that the diagnosis of COPD, present tracheostomy, necessity of blood transfusion, supraglottic tumor location, hypopharyngeal cancer, advanced primary tumors, positive resection margins, and simultaneous neck dissection are associated with a higher incidence of pharyngocutaneous fistulas after total laryngectomy [221], [222], [223].

The development of pharyngocutaneous fistulas is multifactorial; and patients who had received primary radio(chemo)therapy are a specific risk group. The identification of high-risk patients is crucial in order to reduce the possible risk of fistula development by undertaking preoperative measures.

In a meta-analysis of 33 studies, an incidence for pharyngocutaneous fistulas of 34.1% after salvage laryngectomy after previous radiochemotherapy, of 22.8% after salvage laryngectomy after radiotherapy alone, and of 10.3% after salvage laryngectomy with flap insertion was observed [224]. By inserting flaps during salvage laryngectomy, the relative risk for pharyngeal fistulas was reduced to 0.556 [224]. This aspect was confirmed by a systematic review showing that the reconstruction by flaps from a non-irradiated area reduced the risk of pharyngocutaneous fistulas after salvage laryngectomy by one third [225]. According to these data, the insertion of flaps during salvage laryngectomy can be recommended to prevent pharyngocutaneous fistulas.

No consensus is found in the literature about which tissue transfer is the optimal one. Also on this issue nearly exclusively retrospective cohort studies are available. The majority of the studies is found for pectoralis major flaps [226]. Few studies compare two different flaps [227]. A retrospective cohort analysis of 359 patients about pharyngeal fistulas after salvage laryngectomy could show that the rate of fistulas after reconstruction with pectoralis major flaps (15%) was significantly lower than after reconstruction with free flaps (25%) or primary wound closure (34%). In patients who developed fistulas, the persistence of the fistula with primary closure (14 weeks) was significantly longer in comparison to reconstruction with pectoralis major flaps (9 weeks) or free flaps (6.5 weeks) [228].

A prospective randomized study of a small patient population of 19 patients could show a higher complication rate (flap failure, pharyngeal fistula, pharyngeal stenosis) after antero-lateral thigh flap in comparison to radial forearm flap [229]. These data have to be verified in studies with higher case numbers.

The conclusion may be drawn that the insertion of tissue from a non-irradiated area significantly reduces the risk of fistulas after salvage laryngectomy, however, there is no evidence on the type of tissue transfer.

Possibly, robotic laryngectomy will be applied more frequently for salvage situations in the future. The advantage of this technique is that pharyngotomy and external skin incision are clearly smaller due to the transoral preparation, fascia between neopharynx and carotid sheath remain intact. Probably, this reduces the risk of pharyngocutaneous fistulas and carotid artery rupture [65]. First case reports on robotic laryngectomy for larynx cancer recurrences have been published [74], [75], however, the verification of the results in larger populations is expected.

## 5 Evidence regarding the safety margins for resection of laryngeal carcinomas

Since the risk of local recurrence correlates with the resection stage R0 resection is decisive in laryngeal cancer surgery [230]. The exact assessment of the resection margins is a key factor of tumor therapy. If R0 resection is not achieved in a first intervention, a second resection should be performed. The oncological outcome of patients with R0 stage after one or several surgical interventions seems to be comparable [230]. If another surgery is not possible, adjuvant radiation is a possibility to reduce the risk of local recurrences [230].

Currently, there is no consensus about the adequate safety margins in the larynx [231], [232]. A sufficient safety margin is necessary in order to include possible submucous tumor spread [233]. But especially each unnecessary tissue resection on the glottic level may significantly impair the voice function. For supraglottic carcinomas and advanced glottic carcinomas, a safety margin of at least 0.5 cm is required [233], [234] because it became obvious that patients with safety margins of less than 0.5 cm had an outcome that was comparable to patients with R1 resection. Even postoperative radiotherapy of R1 resection or lower safety margins did not lead to the same disease-free survival than sufficient safety margins [235]. Other authors suggested safety margins of at least 1 mm as adequate for small glottic carcinomas [231], [232], [236]. These recommendations are based on retrospective studies. Prospective randomized studies on this question are not available so that a evidence-based recommendation cannot be given. The histological assessment of the resection margins after laser surgery may be complicated by thermal alterations. Additionally, since piecemeal resection of the tumor cannot always be avoided in transoral surgery, definition of the safety margins is only possible when the resections are exactly described regarding their topography.

## 6 Evidence of perioperative antibiotic therapy in the context of laryngeal cancer surgery

The AWMF guideline on perioperative antibiotic prophylaxis recommends that the indication of perioperative antibiotic prophylaxis should depend on the type of surgical intervention, wound classification, individual as well as pre-, intra-, and postoperative risk factors [237]. Individual patient-specific factors that might influence the risk of postoperative wound infection, are for example age, nutritional status, comorbidities such as diabetes mellitus, anemia, and peripheral vascular diseases, but also tobacco abuse, alcohol abuse, and medication [238], [239], [240], [241], [242], [243], [244], [245]. The presence of malignant disease is also a risk factor for the occurrence of wound infection. Antibiosis for more than 24 hours is no longer called prophylaxis, but therapy [237].

Some randomized controlled trials on tumor surgery of the neck could show a higher infection rate after placebo administration compared to antibiotic prophylaxis [246], [247], [248]. The incidence of wound infection after clean-contaminated oncological interventions in the head and neck area without administration of perioperative antibiotic prophylaxis amounts to 30-80% depending on the patient groups [249], [250]. Studies on clean-contaminated interventions of the head and neck, however, do not show any difference regarding the effectiveness of antibiosis for 24 hours and antibiotic therapy for several days [239], [240], [241], [251], [252].

Therapy effective antibiotic levels have to be present at the beginning of surgery so that the first dose of the antibiotic should be given in time before starting surgery [237]. The singular application of an antibiotic agent is considered as effective prophylaxis for durations of surgery of less than 2 hours. For longer surgeries, a subsequent dose should be administered depending on the half-life period of the antibiotic [237].

A clear recommendation for a specific antibiotic or combination that should be applied in the context of clean-contaminated interventions of the larynx cannot be given because of missing evidence. According to a recent analysis, clindamycine should not be applied. Langermann et al. [253] could show in a retrospective analysis of patients after total laryngectomy that for 1,865 hospital admissions 439 different antibiotic schemes were used. Regarding singular application as well as application in combination, the administration of clindamycine was associated with an increased wound infection rate, wound dehiscence, and antibiotic-related complications [253]. One major problem is the missing effect on gram-negative pathogens. This aspect is confirmed by data of the only randomized clinical trial on the comparison of clindamycine with ampicillin sulbactam in the context of oncological head and neck surgery [254].

The risk for wound infection is relevantly increased in salvage surgery. It is currently not proven if long-term antibiotic therapy is appropriate. While some authors favor long-term antibiotic therapy for salvage interventions as salvage laryngectomy [255], [256], [257], other studies consider perioperative antibiotic prophylaxis with 4 doses over 24 hours as equally effective [258], [259]. In an analysis performed by Scotton et al. [260], it could be revealed that the pathogens detected in wound infections after salvage laryngectomy, i.e. MRSA, Pseudomonas aeruginosa, Serratia marcescens, Proteus mirabilis, and Enterococcus faecalis, were pathogens typically acquired in hospitals that were often not covered by antibiotic prophylaxis with teicoplanin, cefuroxime, and metronidazole. Scotton et al. [260] therefore recommended to determine special protocols for antibiotic prophylaxis for patients in the salvage situation after primary radio(chemo)therapy.

## 7 Follow-up after surgical therapy of laryngeal cancer

Important objectives of follow-up after surgical therapy of laryngeal cancer are the early detection of recurrences and secondary tumors. Their incidence during follow-up amounts to constantly 4% per year [261]. Furthermore, the reduction of tobacco and alcohol consumption is relevant. The long-term intention of follow-up is to improve the survival of the patients. Up to now there is no evidence that regular follow-up of patients with laryngeal cancer leads to a better overall survival [262]. In 2014, the European Laryngological Society published a total of 21 recommendations for follow-up of patients with laryngeal cancer [262]. It is recommended to perform regular follow-up examinations for at least 5 years after first diagnosis, while 8 weeks intervals in the first 2 years and afterwards 3–6 months intervals should be observed. Patients with tumors of stage IV should undergo follow-up examinations for 10 years and patients with secondary carcinomas should even present life-long for follow-up examination. Among those 21 recommendations that are taken from the publication, 4 recommendations meet grade A criteria. Three of them concern the necessity of imaging after primary radio(chemo)therapy or multimodal treatment. The 4^th^ recommendation of grade A evidence contains the patients’ training. It is recommended that patients are informed about typical symptoms and hints of recurrences and to submit the patients to non-smoking and non-drinking programs [262], [263]. This recommendation is justified by the statement that the knowledge of possible symptoms is a key factor for the early diagnosis of recurrences or secondary carcinomas [264]. Patients should furthermore have the possibility to contact the physicians involved in the follow-up in order to undergo immediate examinations in cases of typical symptoms [262].

## 8 Conclusion and outlook

Regarding surgical procedures for therapy of laryngeal cancer, scientific knowledge of high evidence as it is gained by randomized controlled trials is missing. For most interventions, there are larger case series and sometimes also comparisons of prospectively performed investigations with a historical control group; however, according to criteria of evidence-based medicine the data are not sufficient to give evidence-based recommendations for a surgical option.

The reasons for this fact are manifold. The conduction of studies on surgical interventions is complex, especially the reproducibility of surgical results gained in randomized studies is more difficult than in clearly defined protocols of pharmacological trials where pharmaco-dynamic parameters can be easily measured. A real standard of surgical performance is difficult to achieve because surgery is always associated with the experience and the technical skills of the surgeon who mostly has a personal preference for certain surgical procedures. Even old and new surgical methods are difficult to compare because the experience of the surgeon has an enormous impact on the success of the intervention and a reliable surgical outcome can only be achieved after a learning curve. This could be shown for transoral laser surgery of laryngeal carcinomas [265]. So when comparing two surgical procedures, it is important that the surgeons have the same expertise in both surgical procedures because otherwise a bias of the study results is found. An additional factor is certainly the difficulty of the patients to subject to randomization regarding such a vital therapy. This problem can only be overcome if the informing physician is fully convinced of the randomization.

One problem of the conducted studies are also the different target parameters such as progression-free survival, disease-free survival, disease-specific survival, laryngectomy-free survival, overall survival, and different measurement instruments for assessing functional results. This limits systematic analysis and comparability of data of different primary studies.

The mentioned problems lead to the situation that only a small percentage of laryngeal cancer surgeries is justified by randomized study data. An ENT surgeon has to face the difficulty that retrospective cohort studies on surgical procedures have to be compared with randomized controlled prospective trials on different radiochemotherapy protocols. Hereby non-surgical therapeutic procedures have gained in importance in the last decades; this development was further promoted by the introduction of antibody and immune therapy. On the other hand, recent studies lead to a new assessment of primary surgical concepts. Large retrospective studies of T4a laryngeal carcinomas revealed a superiority of total laryngectomy and postoperative radiotherapy compared to multimodal organ-preserving therapy concerning survival [141] and late functionality [142]. Those data contradict to an unreflected laryngeal preservation.

Shaw et al. [266] expect a renaissance of surgical studies in the field of head and neck oncology. Especially in the Anglo-American countries numerous studies on surgical therapeutic concepts have been initiated [266]. This aspect was also promoted by an orientation to transoral robotic surgery in the US. Further knowledge regarding laryngeal cancer is expected from clinical studies such as the SUPRATOL study, but also other trials on surgical therapies that are listed under clinicaltrials.gov. These studies may lead to a better evidence regarding laryngeal cancer surgery.

## Notes

### Competing interests

The author declares that he has no competing interests.

### Acknowledgements

Special thanks to Prof. Dr. Andreas Dietz and Prof. Dr. Jochen A. Werner for their estimated comments on this article.

## Figures and Tables

**Table 1:**
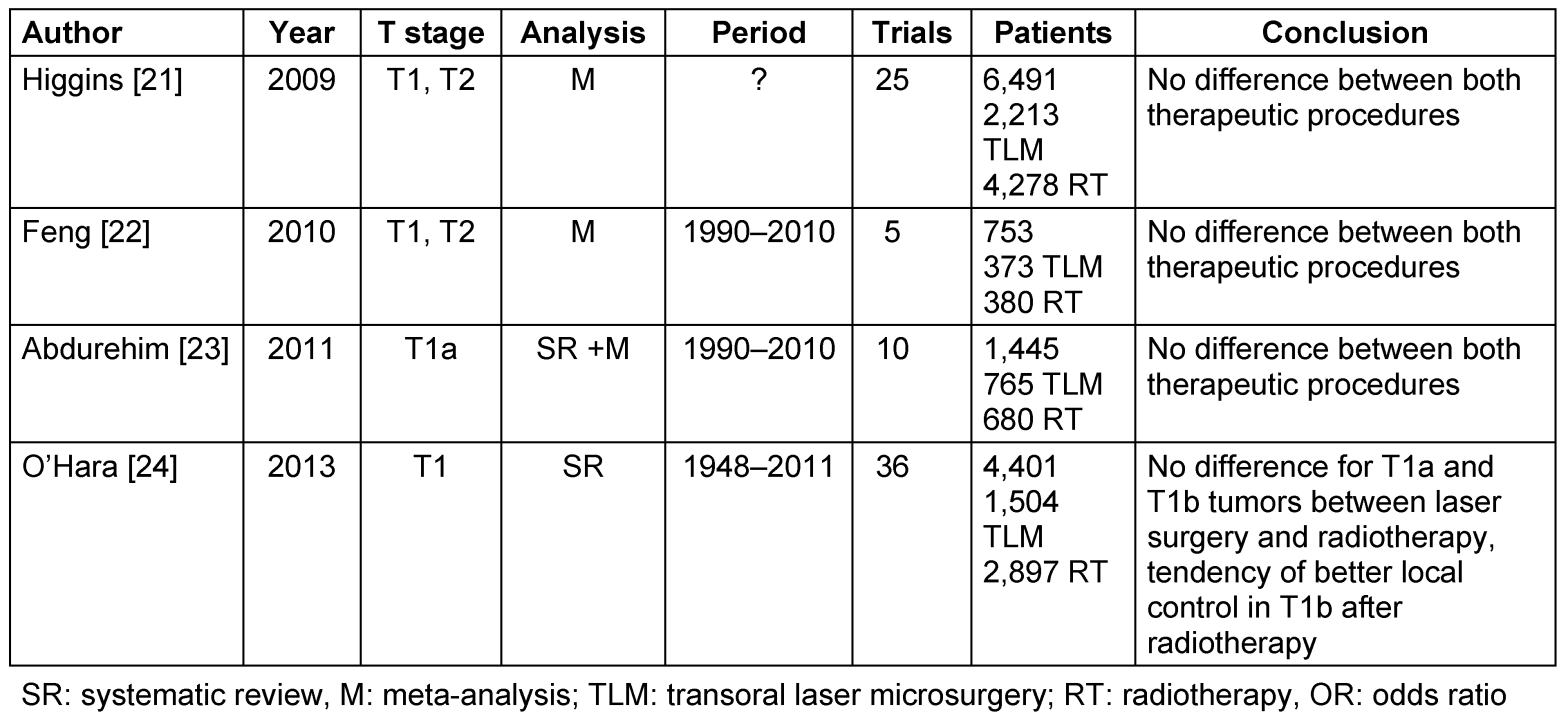
Meta-analyses and systematic reviews comparing the local control after radiotherapy and transoral laser surgery of T1 and T2 glottic carcinomas

**Table 2:**
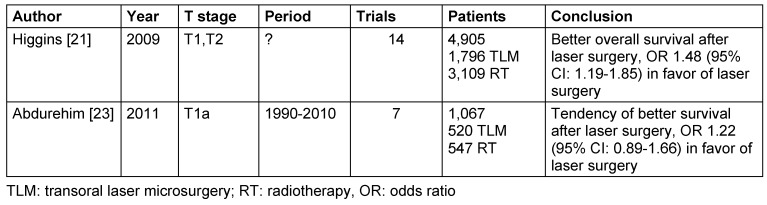
Meta-analyses comparing the overall survival after radiotherapy and transoral laser surgery of T1 and T2 glottic carcinomas

**Table 3:**
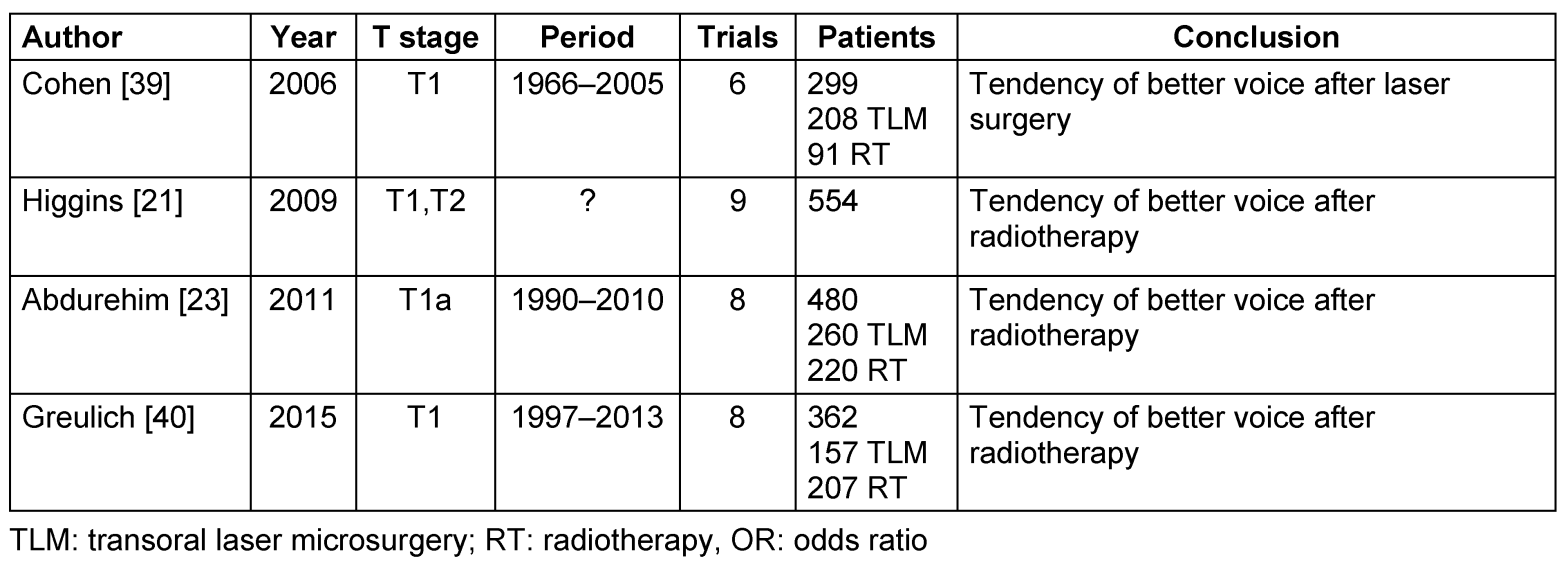
Meta-analyses comparing the voice quality after radiotherapy and transoral laser surgery of T1 and T2 glottic carcinomas

**Table 4:**
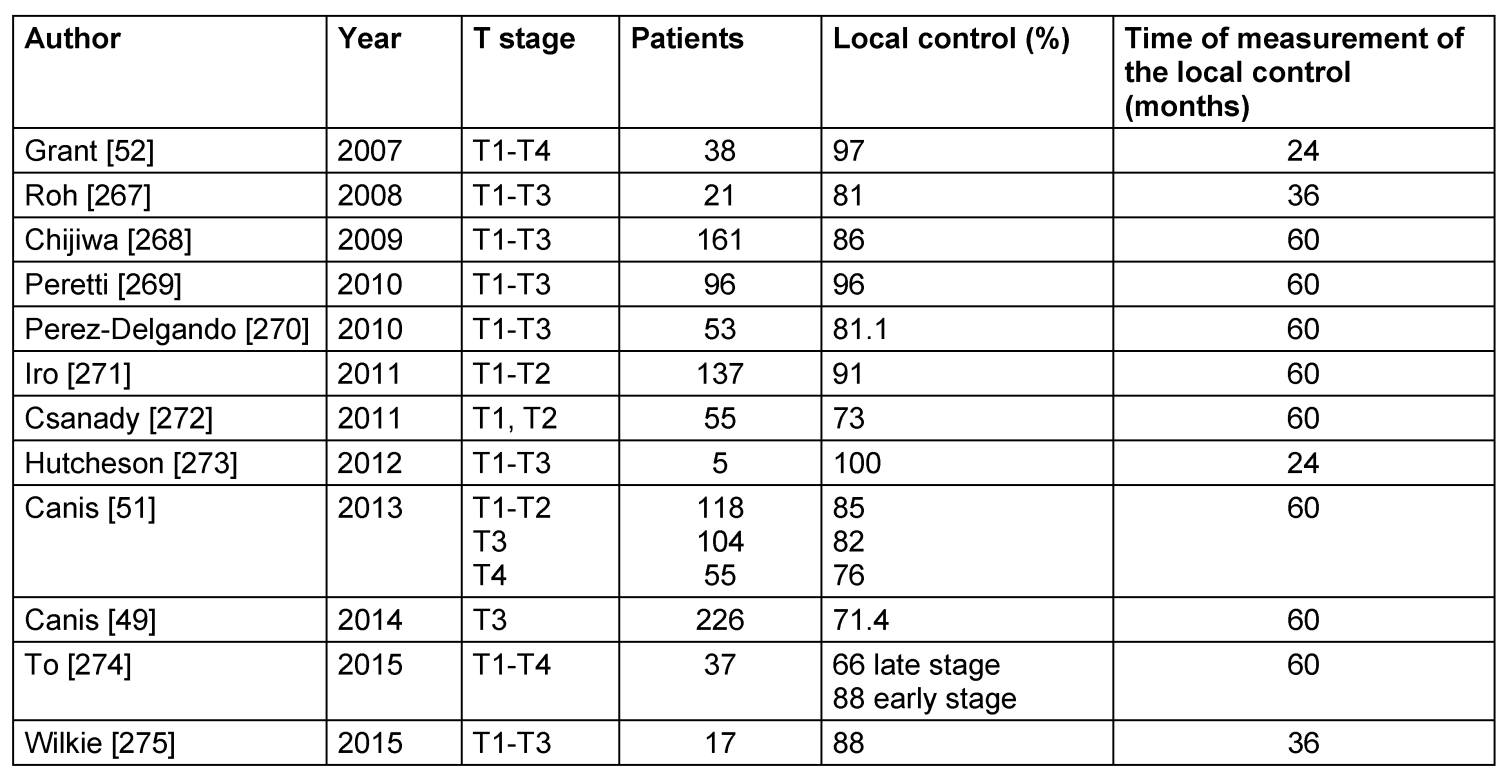
Studies on the local control after transoral supraglottic partial laser resection of the last 10 years

**Table 5:**
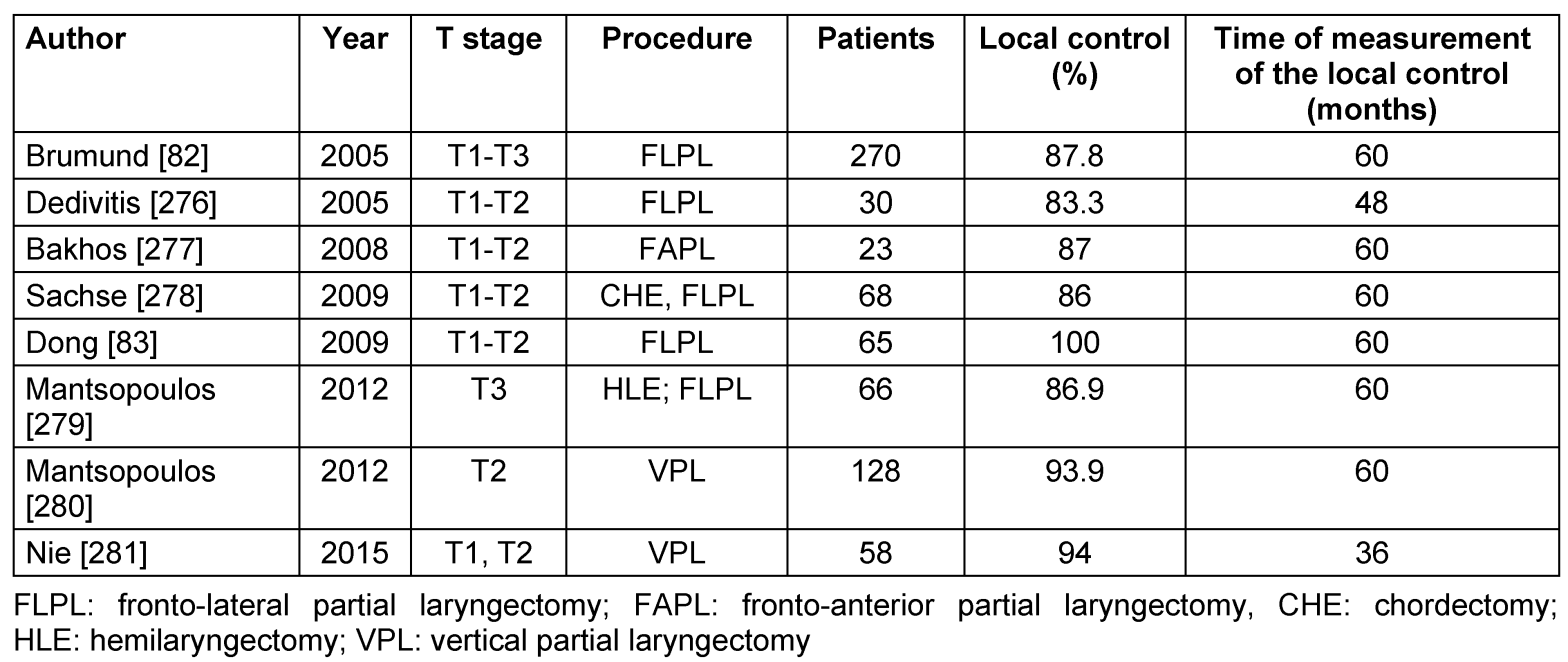
Studies on the local control after open vertical partial laryngectomy of the last 10 years

**Table 6:**
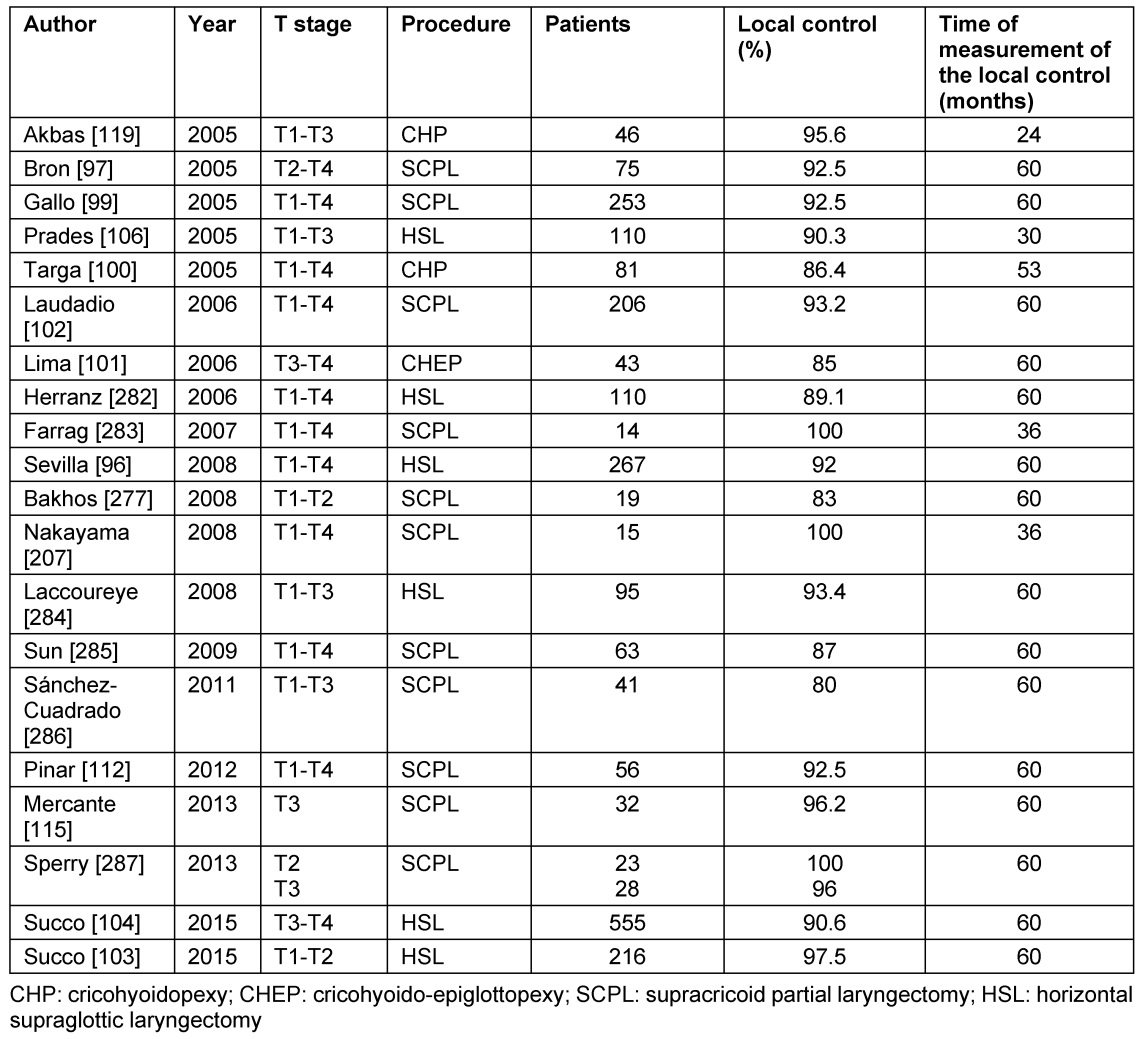
Studies on the local control after open horizontal partial laryngectomy of the last 10 years
